# Active Support Correction and Variable Triangular Layout Optimization for an Annular Thin Mirror in Optical Remote-Sensing Systems

**DOI:** 10.3390/s26175366

**Published:** 2026-08-25

**Authors:** Lanxin Peng, Changzheng Chen

**Affiliations:** 1 Changchun Institute of Optics, Fine Mechanics and Physics, Chinese Academy of Sciences, Changchun 130033, China; penglanxin21@mails.ucas.ac.cn; 2 University of Chinese Academy of Sciences, Beijing 100049, China

**Keywords:** active optics, annular thin mirror, active support, variable triangular layout, Annular Zernike polynomials, Kriging surrogate model, influence function, CMA-ES

## Abstract

Surface-figure stability is essential to high-resolution optical remote-sensing systems that employ lightweight annular primary mirrors. This study proposes a variable triangular active support layout for a 660 mm annular thin mirror with a 100 mm central aperture. The layout is parameterized within a one-sixth annular sector and constructed over the full aperture by six-fold rotational replication. Finite-element influence functions, Annular Zernike modal fitting, and Kriging surrogate modeling are integrated to assess modal controllability while reducing the cost of repeated finite-element analyses. The objective function accounts for surface-figure maintenance under gravity, correction residuals for representative low-order Annular Zernike modes, geometric constraints, and the actuator-force limit. Optimization is performed using the covariance matrix adaptation evolution strategy (CMA-ES), followed by local refinement. For a 36-point support configuration, the optimized variable triangular layout satisfies the 12.66 nm RMS residual requirement under axial and radial gravity and for all representative *Z*_4_–*Z*_11_ target surfaces. Among the annular, triangular, square, hexagonal, Fibonacci, and proposed layouts, the proposed layout yields the lowest composite objective-function value, a mean corrected RMS residual of 2.15 nm, and an effective response-matrix rank of 35. Monte Carlo simulations with independent ±1 mm support-position perturbations further demonstrate its robustness to installation errors.

## 1. Introduction

Space remote-sensing, astronomical-observation, and high-resolution imaging systems increasingly require larger apertures, lower structural mass, and greater surface-figure stability. In these systems, low-order primary-mirror figure errors caused by gravity, thermal loads, manufacturing residuals, and alignment errors can degrade imaging performance. Increasing the mirror thickness and support stiffness is a conventional solution, but it also increases mass, manufacturing complexity, and launch or installation costs. Active optics maintains the desired surface figure of a flexible or lightweight primary mirror through wavefront sensing, controlled mirror deformation, and optical-element pose adjustment. It has therefore become a key technology for large ground-based telescopes and next-generation spaceborne optical payloads [1,2,3].

The central problem in an active-optics system is to establish the mapping from actuator commands to the resulting mirror response. Wilson et al. showed that primary-mirror figure and alignment errors can be decomposed into low-order wavefront modes and corrected through feedback control [1]. Noethe reviewed the wavefront-analysis methods, correction mechanisms, and engineering applications of active optics in modern large telescopes [2]. Practical systems such as the VLT Survey Telescope (VST) have demonstrated low-order aberration correction using combined axial and lateral active supports [4]. For active thin mirrors, the actuator influence functions and support topology determine how completely the target modal space can be spanned. Previous studies of annular mirrors, actuator influence functions, and optimized axial-support topologies provide the foundation for support-layout design [5,6,7].

Recent research on active support layout optimization has progressed from minimizing gravity-induced deformation alone to multiobjective, constrained, and controllability-oriented design. Zhou et al. developed a whiffletree-supported active-optics scheme for space applications [8]. Dai et al., Li et al., and Yu et al. investigated active correction and support systems for 1.2 m and 4 m primary mirrors, including a 4 m SiC mirror, and validated influence-function measurement, Zernike fitting, gravity compensation, and bending-mode correction [9,10,11]. More recently, Buffo et al. compared measured actuator influence functions with FE predictions for an adjustable thin X-ray mirror and demonstrated figure correction using distributed piezoelectric actuation [12]. For surface-figure representation, Mahajan applied Gram–Schmidt orthogonalization to extend the Zernike basis to annular domains, thereby avoiding the loss of orthogonality and coefficient coupling that occur when circular-pupil Zernike polynomials are applied directly to an annular aperture [13]. Annular Zernike polynomials are well suited to representing the global surface figure of a circular mirror with a central obscuration and correspond directly to conventional optical aberrations, including defocus, astigmatism, coma, and spherical aberration [14]. Forbes’s Q-polynomials provide improved numerical robustness and coefficient convergence for steep aspheric and freeform surfaces and are therefore more appropriate for surface parameterization during optical design [15,16,17]. By contrast, B-splines and other localized bases efficiently represent mid- and high-spatial-frequency features, such as support print-through, using piecewise polynomials and control points [18]. Related localized and orthogonal bases have also been applied successfully to wavefront representation, wavefront-shaping control, and high-accuracy local fitting of optical surfaces [19,20,21].

Obtaining unit-force responses at candidate support locations requires repeated finite-element (FE) analyses and is therefore computationally expensive. The design and analysis of computer experiments, surrogate modeling of complex simulations, and efficient global optimization of black-box functions provide a theoretical basis for response-surface construction and layout optimization [22,23,24]. Recent optical-mirror studies have combined FE sampling with Kriging or neural-network surrogates to optimize structural parameters, support locations, and surface-figure performance [25,26]. For large segmented and monolithic mirrors, shape control, control-matrix construction, force optimization, bending-mode analysis, and constrained least-squares correction now constitute a well-established methodology [27,28,29,30,31,32]. Studies of axial-force errors, highly degenerate active-optics systems, and honeycomb mirrors also provide useful guidance on error propagation and robust correction [33,34,35].

The correction capability of an active thin mirror depends strongly on the spatial arrangement of its support points. For an annular mirror, the radial coordinate, azimuthal phase, and local connectivity of each support determine the spatial characteristics of the unit-force influence functions. These characteristics, in turn, govern the projection residual in the influence-function space, the Annular Zernike fitting residuals, and the singular-value spectrum of the response matrix. Despite substantial progress in active-optics theory, influence-function modeling, thin-mirror correction experiments, and support-system engineering, two challenges remain. First, conventional annular and fixed-lattice layouts may contain weakly controllable modes and exhibit local force amplification when the actuator count is limited. Second, a fully free layout may improve theoretical performance but complicates assembly and alignment. Whereas recent surrogate-assisted studies have optimized prescribed structural or support-ring parameters [25,26], the present study optimizes local support positions within a topology-preserving triangular network and evaluates the resulting layouts in the Annular Zernike controllability space. Active support layout optimization is thus treated as a distinct optomechanical design problem for annular thin mirrors.

This study considers a 660 mm aperture annular thin mirror with a 100 mm central aperture. A six-sector variable triangular support layout is proposed to balance modal controllability, structural symmetry, installation spacing, and optimization dimensionality. Its overall correction capability is evaluated for gravity-induced deformation and representative low-order Annular Zernike aberrations.

## 2. Problem Definition and Evaluation Metrics

**Figure 1:**
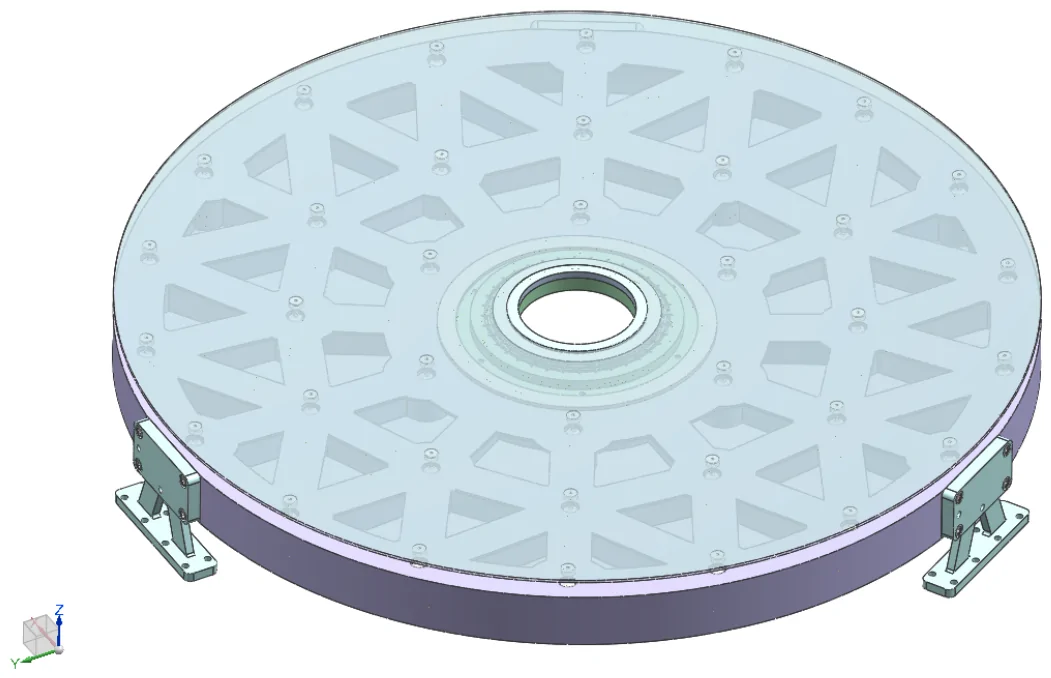
Support configuration of the annular thin mirror.

The active thin-mirror system combines a passive central flexure with discrete axial actuators. The central flexure constrains the $T_{X}$, $T_{Y}$, and $R_{Z}$ degrees of freedom, whereas the active supports constrain $T_{Z}$, $R_{X}$, and $R_{Y}$. Figure 1 shows the resulting support configuration.

The design variables are the positions of the discrete axial supports on the rear surface of the annular mirror, with each actuator applying an axial correction force. The objective is to span the gravity-deformation and representative low-order aberration spaces as effectively as possible for a fixed support count, allowable layout domain, minimum installation spacing, and actuator-force limit. The evaluation targets comprise surface-figure maintenance under gravity and correction of the low-order Annular Zernike modes *Z*_4_–*Z*_11_. Each *Z*_4_–*Z*_11_ case represents the maximum correction demand considered when the corresponding aberration occurs independently. The reference wavelength *λ* is 632.8 nm, corresponding to a He–Ne laser, so 0.02*λ* is approximately 12.66 nm. The Zernike modes follow the Noll indexing convention, and all RMS values refer to mirror surface-figure errors. Table 1 summarizes the target surfaces, prescribed amplitudes, and residual requirements.

The unified evaluation also includes layout constraints. All supports must remain within the allowable annular region, satisfy the prescribed minimum spacing, and operate within the actuator-force limit. Rotational symmetry is retained to facilitate actuator grouping and alignment.

## 3. Correction Mechanism of Multipoint Active Support

Active correction of the thin mirror is modeled using linear superposition and modal decomposition. Let w(R,θ) denote the target surface, $g_{i}\left(R,\theta ;R_{i},\theta_{i}\right)$ the unit-force influence function of the *i*th support at $\left(R_{i},\theta_{i}\right)$, and $f_{i}$ its correction force. The surface generated by the active supports is then given by Equation (1):

(1) $$w_{c}\left(R,\theta \right)=\sum_{i=1}^{N}f_{i}\;g_{i}\left(R,\theta ;R_{i},\theta_{i}\right).$$

The target surface is expanded in the Annular Zernike basis $\phi_{q}\left(R,\theta \right)$ as shown in Equation (2):

(2) $$w\left(R,\theta \right)=\sum_{q=1}^{Q}a_{q}\phi_{q}\left(R,\theta \right),$$

where $a_{q}$ denotes the coefficient of the *q*th target mode. The Annular Zernike basis functions $\phi_{q}$ are constructed by applying Gram–Schmidt orthogonalization to the circular Zernike polynomials over the annular pupil, following Mahajan’s formulation [13], and are normalized to unit RMS over the annular aperture. Equation (3) defines the modal response matrix:

(3) $$H_{qi}=\langle \phi_{q},g_{i}\rangle .$$

The target coefficients and actuator forces are related by Equation (4):

(4) a≈Hf.

The unconstrained least-squares solution is given by Equation (5):

(5) $$f_{\mathrm{opt}}=H^{+}a,$$

where $H^{+}$ denotes the Moore–Penrose pseudoinverse [38]. The minimum projection residual outside the subspace spanned by the actuator influence functions is defined by Equation (6):

(6) $$\begin{array}{cc} e_{\min} & =∥a-Hf_{\mathrm{opt}}{∥}_{2}\\ & =∥a-HH^{+}{a∥}_{2},\;P_{H}=HH^{+}. \end{array}$$

Consequently, correction capability depends on how completely the response matrix spans the target modal space. The layouts are therefore evaluated in terms of Annular Zernike fitting residuals, effective rank, retention of the tail singular values, and actuator-force demand under the prescribed constraints.

## 4. Factors Affecting Multipoint Support

### 4.1. Basic Mirror Parameters

**Figure 2:**
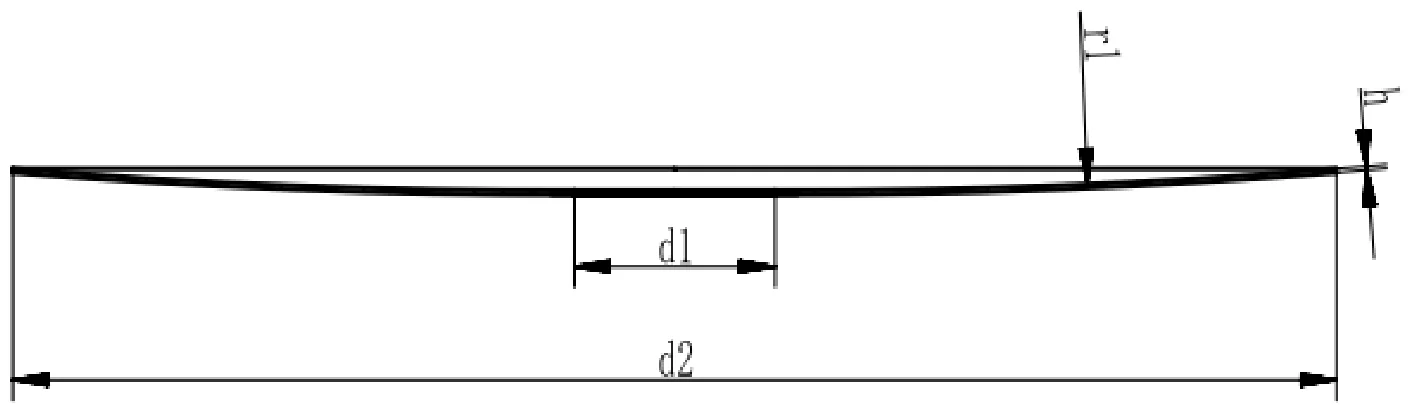
Geometric parameters of the annular thin mirror.

The mirror has a central-aperture diameter of *d*_1_ = 100 mm, a clear-aperture diameter of *d*_2_ = 660 mm, an obscuration ratio of 0.1515, and a radius of curvature of 3300 mm. Its geometry is shown in Figure 2.

The mirror material determines its elastic modulus, Poisson’s ratio, density, coefficient of thermal expansion, and thermal stability. Unlike a passive high-stiffness mirror, an actively supported thin mirror does not necessarily benefit from the highest possible elastic modulus. Provided that the gravity-deformation, natural-frequency, and stress requirements are satisfied, lower out-of-plane bending stiffness increases the displacement produced by a unit correction force and can improve correction efficiency. The mirror thickness governs both the bending stiffness and the unit-force response amplitude, whereas the support-pad diameter determines the local load-transfer area and affects the response and stress near each support. The layout then determines how the individual influence functions sample and combine over the mirror surface. Table 2 lists the candidate materials. On the basis of manufacturability, thermal stability, and active-correction requirements, a Zerodur-like low-CTE glass ceramic is selected for the subsequent analyses.

Mirror thickness directly affects the bending stiffness. To isolate changes in the spatial form of the influence function from simple amplitude scaling, the force applied to each thicker mirror is scaled so that its peak response matches that of the reference mirror. With *h* = 1.6 mm as the reference, the RMSE between the scaled influence functions increases only from 0.183% at *h* = 2.0 mm to 0.772% at *h* = 4.0 mm. The compensated influence functions therefore remain nearly coincident over the thickness range of 1.6–4.0 mm. Within this range, thickness primarily scales the response amplitude and has little effect on the spatial form of the influence function, consistent with the thin-plate bending relation in Equation (7):

(7) $$w\propto \frac{F}{h^{3}},\;\frac{F(h)}{F(1.6)}\approx {\left(\frac{h}{1.6}\right)}^{3}.$$

**Figure 3:**
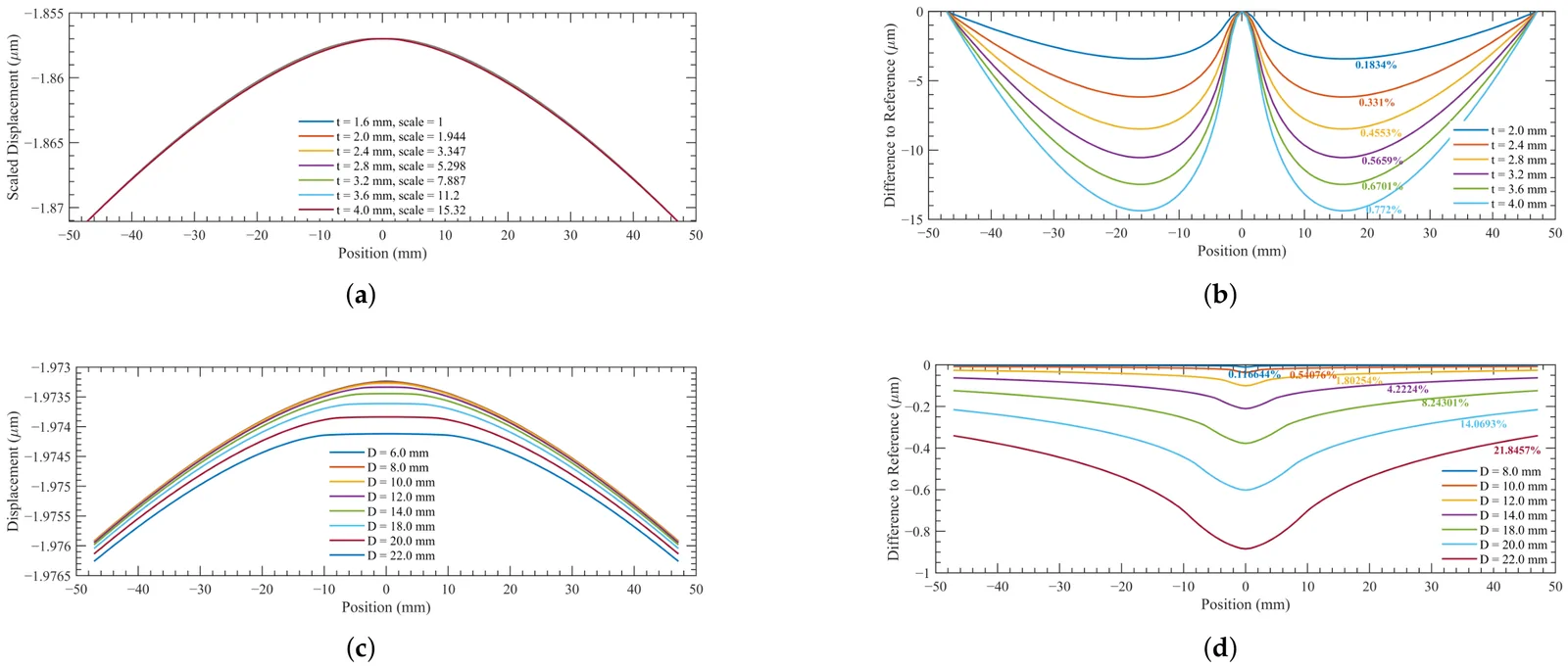
Effects of mirror thickness and support-pad diameter on the influence function: (**a**) force-scaled influence functions for different mirror thicknesses; (**b**) deviations from the 1.6 mm reference-thickness influence function; (**c**) influence functions for different support-pad diameters; and (**d**) deviations from the 6 mm reference-pad influence function.

The support-pad diameter is evaluated in the same manner. With *D* = 6.0 mm as the reference, the influence-function RMSE increases from 0.117% at *D* = 8.0 mm to 21.8% at *D* = 22.0 mm. Over the range of 6.0–22.0 mm, the pad diameter primarily affects the local response near the support and has little influence on the broader response distribution. A smaller pad more closely approximates a point load and is therefore preferable for controlling mid- and high-order surface components. Subject to the mirror-stress requirement, a pad diameter of 6 mm is adopted in the subsequent layout calculations. Figure 3 compares the influence functions and their deviations for the different mirror thicknesses and support-pad diameters.

### 4.2. Typical Support Layouts

In an active-optics system, surface correction can be viewed as approximating a continuous mirror surface with a finite set of actuator influence functions. Correction capability therefore depends not only on the actuator count but also on how the layout samples the spatial frequencies of the target aberration space. The low-order aberrations considered for the annular thin mirror are dominated by large-scale bending patterns, whose controllability depends primarily on the radial positions, azimuthal distribution, and coupling of neighboring supports. An effective influence-function matrix should consequently provide low Annular Zernike residuals, a nondegenerate singular-value spectrum, actuator forces within the specified limit, and stable performance under force constraints.

The reference configurations comprise triangular (TRI), square (SQ), hexagonal (HEX), annular (ANN), and Fibonacci-spiral (FIB) layouts. TRI, SQ, and HEX are truncated regular lattices that facilitate manufacturing, installation, and adjustment. ANN conforms naturally to the annular boundary and is compatible with ring-based supports, whereas FIB provides nonperiodic, approximately uniform sampling.

The TRI layout is formed by truncating a regular triangular lattice. Its supports extend along three lattice directions, providing efficient local coverage and dense nearest-neighbor connectivity. These characteristics reduce unsupported spans and provide a strong response to surface errors with three- or six-fold symmetry. However, truncation by a circular or annular boundary leaves distinct principal directions that can create directional load paths and do not conform fully to the continuous rotational symmetry of an annular mirror.

The SQ layout is based on a Cartesian grid and is straightforward to parameterize, manufacture, and align. It is well suited to deformable mirrors with rectangular or nearly rectangular apertures and orthogonal directional features. For circular or annular mirrors, however, the grid does not conform to the rotationally symmetric boundary and can introduce directional bias. Its response is generally stronger along the coordinate axes than along the diagonals, which can limit the correction of certain azimuthal modes.

The HEX layout has six-fold symmetry and greater local uniformity than the SQ layout. Its in-plane isotropy more closely approximates uniform sampling over a circular aperture, and it generally performs well among regular lattices. Nevertheless, its radial layers remain fixed by the lattice geometry. For annular mirrors, this radial sampling may be insufficient to correct mid- and high-order aberrations with pronounced radial variation.

The ANN layout distributes supports over several concentric rings. It conforms naturally to circular and annular boundaries and integrates readily with ring-based support structures and radial load paths. It represents low-order axisymmetric modes and some low-order nonaxisymmetric modes, such as defocus and astigmatism, effectively. Its principal limitation is sparse radial sampling: with only a few rings, the supports occupy a small number of discrete radii and cannot independently represent multiple radial orders. Correction performance therefore deteriorates for spherical aberration, higher-order trefoil, and other modes with substantial radial oscillation.

**Figure 4:**
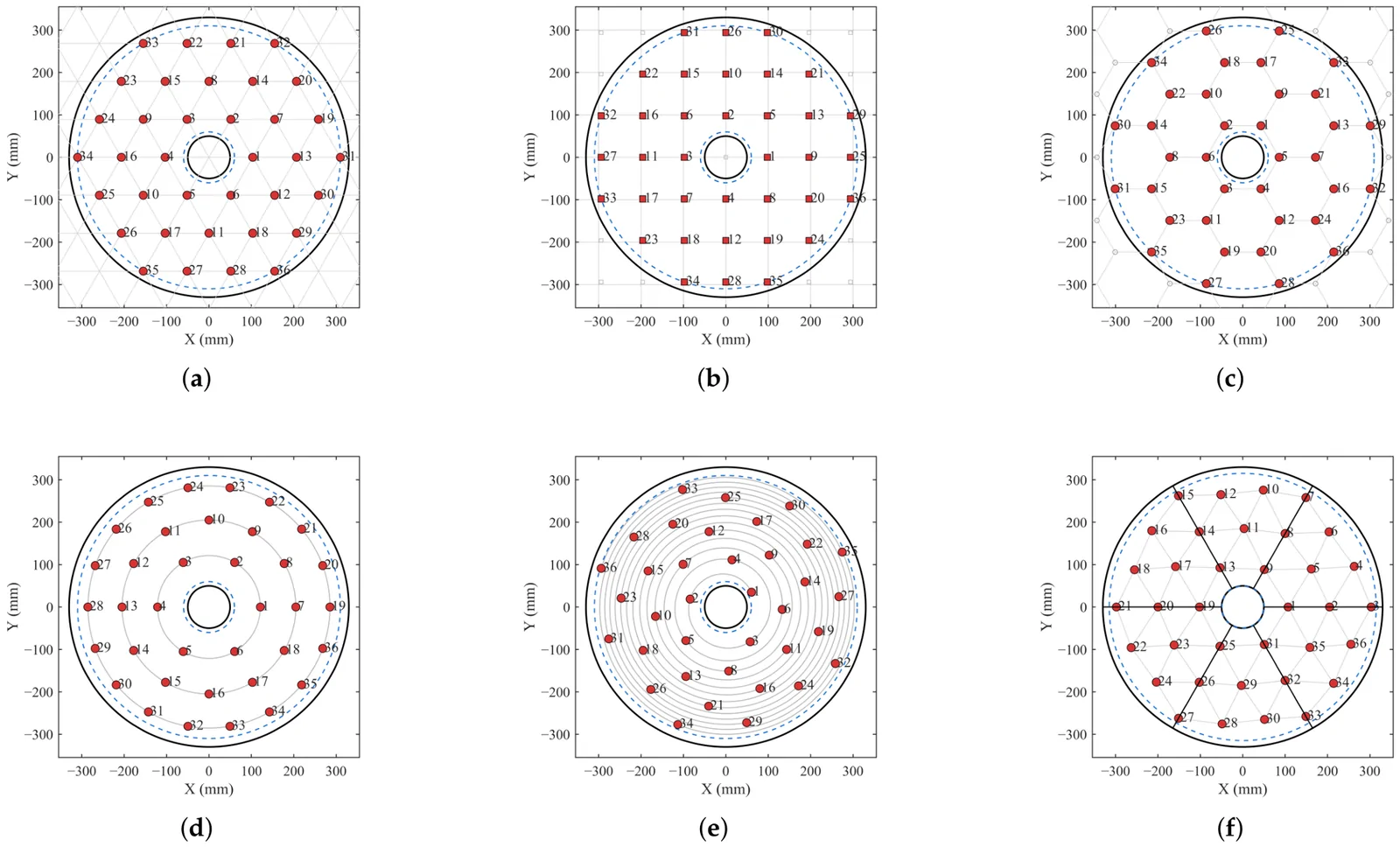
Typical support layouts: (**a**) TRI; (**b**) SQ; (**c**) HEX; (**d**) ANN; (**e**) FIB; and (**f**) VTRI.

The FIB layout follows a spiral rule without fixed rings or azimuthal phases. Its rich radial and azimuthal sampling can increase the effective rank of the response matrix, reduce modal coupling, and improve the correction of several low- and mid-order aberrations. The absence of regular rings and well-defined secondary symmetry, however, complicates hard-point placement, actuator grouping, alignment referencing, and mechanical integration. FIB is therefore more suitable for theoretical comparison and topology optimization than for standardized engineering implementation. Figure 4 compares the six layout geometries, and Table 3 summarizes their symmetry and parameterization characteristics.

Support-layout optimization is therefore not simply a matter of increasing the number of supports. Its purpose is to improve the ability of the influence-function matrix to span the target aberration space with a limited number of actuators. An effective layout for an annular thin mirror must balance local support efficiency, compatibility with the aperture boundary, radial and azimuthal sampling, modal controllability, and engineering feasibility.

### 4.3. Variable Triangular Support Layout

To combine the manufacturability of a regular lattice with the modal adaptability of a free layout, a variable triangular support layout (VTRI) is proposed. A one-sixth annular sector serves as the basic design domain. A regular triangular grid is first constructed within the sector, after which its nodes are allowed to move locally subject to radial-boundary, sector-boundary, minimum-spacing, and mesh-quality constraints. The complete support array is then generated by 60° rotational replication. Unlike TRI, SQ, and HEX, whose geometries are governed primarily by lattice spacing and global rotation, VTRI treats the radial and azimuthal coordinates of the local nodes as design variables. In contrast to a fully free layout, VTRI retains six-sector rotational symmetry and triangular connectivity, allowing engineering constraints to be imposed directly through the network topology.

**Figure 5:**
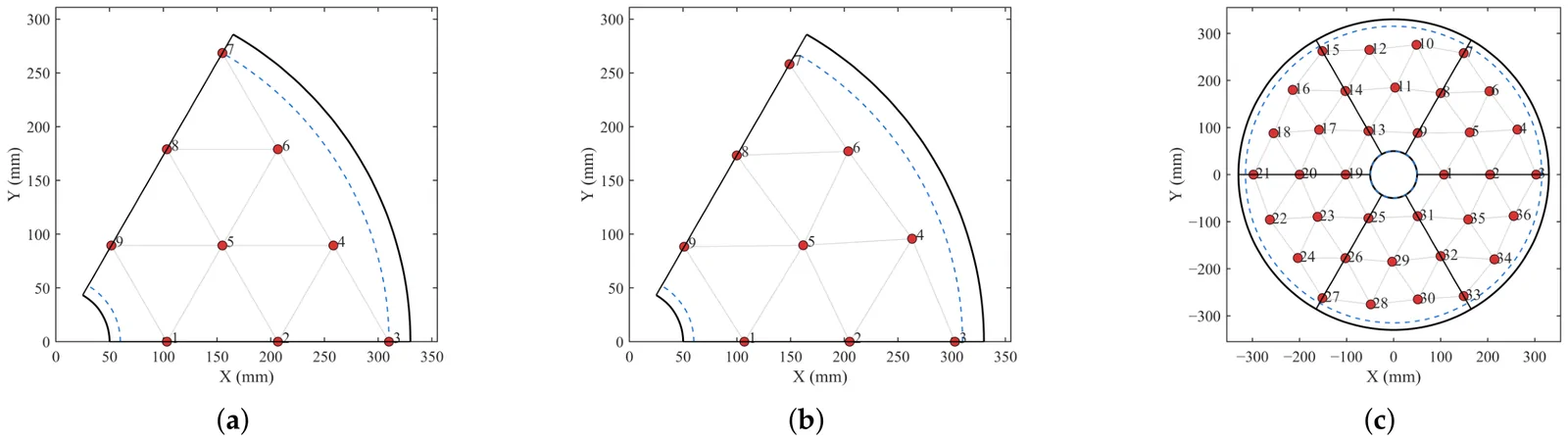
Generation of the VTRI layout: (**a**) initial triangular grid in the basic sector; (**b**) optimized variable triangular grid; and (**c**) full layout generated by six-fold rotational replication.

Figure 5 summarizes the three-stage construction procedure. First, a regular triangular grid is generated within the one-sixth annular sector and clipped at the inner and outer support boundaries, yielding the initial feasible nodes shown in Figure 5a. Second, these nodes are optimized subject to radial-boundary, angular-range, minimum-spacing, and triangular mesh-quality constraints, producing the deformed grid in Figure 5b. Throughout this process, the nodes remain within the allowable sector and preserve their triangular adjacency. Finally, the optimized nodes and edges are replicated about the mirror center at 60° intervals to form the full-aperture layout shown in Figure 5c.

Three geometric constraints are imposed. The independent nodes are confined to the basic sector from 0° to 60°; the minimum internal angle of every triangular element must be at least 40° to prevent slender elements and local support degeneration; and every triangular edge must exceed 50 mm to preserve assembly clearance. Six-sector symmetry is adopted for both optical and engineering reasons. Triangular and hexagonal local patterns naturally exhibit 60° rotational periodicity, and the six-sector construction preserves the local uniformity and in-plane isotropy of the triangular network. The targets comprise gravity-induced deformation and the low-order Annular Zernike modes *Z*_4_–*Z*_11_, including defocus, astigmatism, coma, trefoil, and primary spherical aberration. Six-fold symmetry provides balanced sampling of axisymmetric, two-fold, and three-fold components without giving disproportionate emphasis to trefoil, as may occur with a three-sector layout. Increasing the number of sectors would strengthen symmetry but reduce the design freedom within each sector, driving the layout toward an annular or otherwise highly constrained configuration. Conversely, weakly symmetric and fully free layouts offer larger search spaces but substantially increase the complexity of actuator integration, component standardization, and on-orbit calibration. The six-sector VTRI layout thus provides a compromise between the manufacturability of a regular lattice and the modal adaptability of a free layout.

### 4.4. Theoretical Modal Controllability

For each layout, the support coordinates are generated from the corresponding parameterization. The unit-force influence functions are decomposed into Annular Zernike coefficients to form the response matrix $H\in R^{36\times N}$. Its modal coverage is evaluated through the singular-value decomposition in Equation (8):

(8) $$\begin{array}{cc} H & =U\Sigma V^{T},\\ \Sigma & =\mathrm{diag}(\sigma_{1},\sigma_{2},\ldots ,\sigma_{36}),\\ \sigma_{1} & \geq \sigma_{2}\geq \cdots \geq \sigma_{36}\geq 0. \end{array}$$

Each singular value represents the control gain along the corresponding orthogonal modal direction. Slow singular-value decay and a large minimum effective singular value indicate greater gain in weakly controllable directions, more balanced modal coverage, and more complete linear controllability. Conversely, a rapidly decaying tail or near-zero singular values identify modal combinations that are difficult to excite and therefore reveal layout-induced limitations.

**Figure 6:**
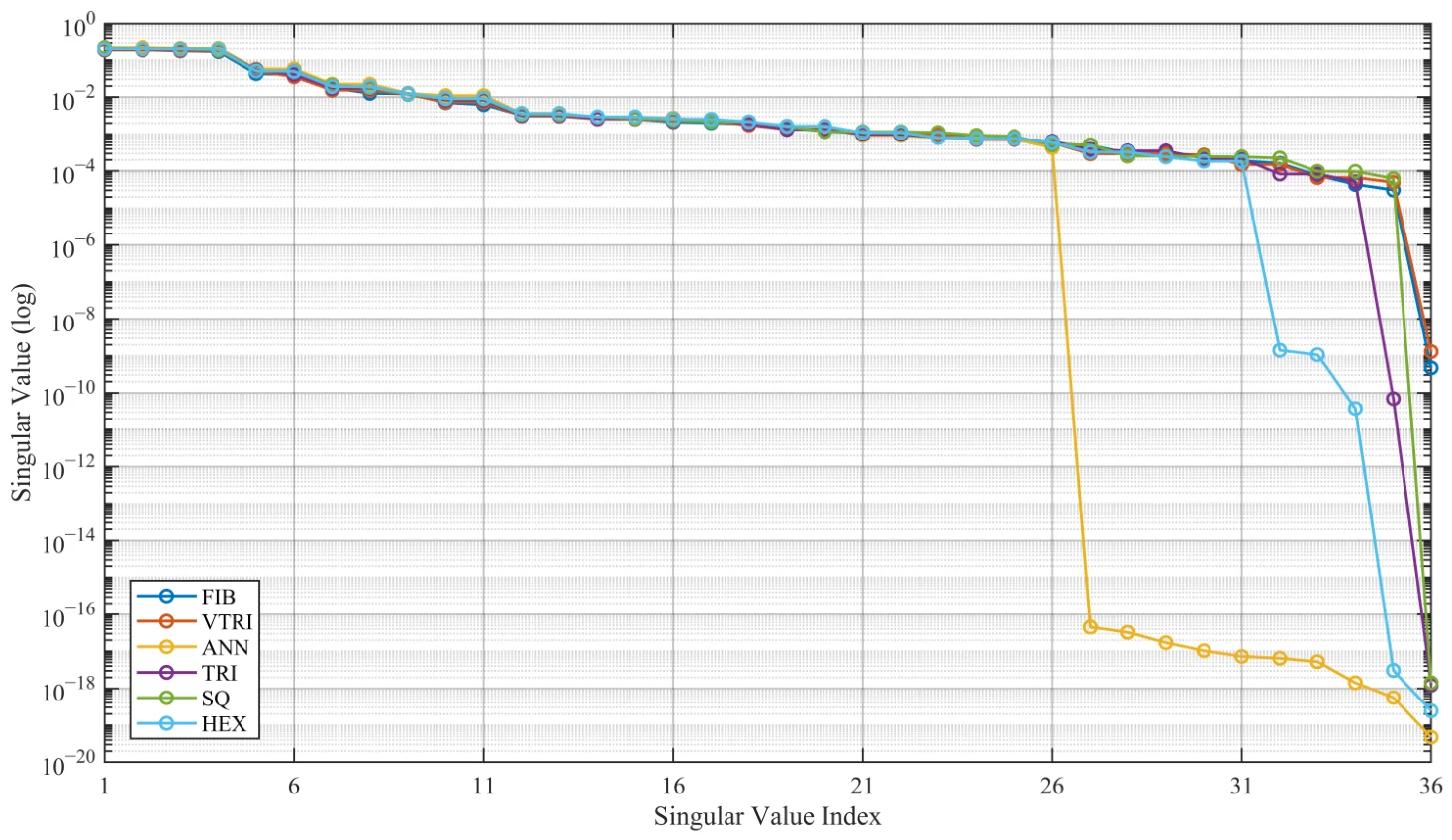
Singular-value spectra of response matrices for different support layouts.

As shown in Figure 6, the leading singular values are similar for all six layouts, indicating comparable control authority in the dominant modal directions. The principal differences occur in the middle and tail of the spectra, particularly in the minimum effective singular value and the emergence of near-rank-deficient directions. ANN responds strongly to low-order, nearly axisymmetric modes, but its spectrum decays rapidly in several combined-modal directions, indicating limited target-space coverage. TRI, SQ, and HEX improve on ANN but remain affected by lattice symmetry and boundary truncation. VTRI raises the lower bound of the tail singular values and reduces the modal degeneracy associated with annular and fixed-lattice layouts; it is, therefore, selected for subsequent optimization.

## 5. Finite-Element and Surrogate Models

### 5.1. Finite-Element Model and Equivalent Boundary Conditions

**Figure 7:**
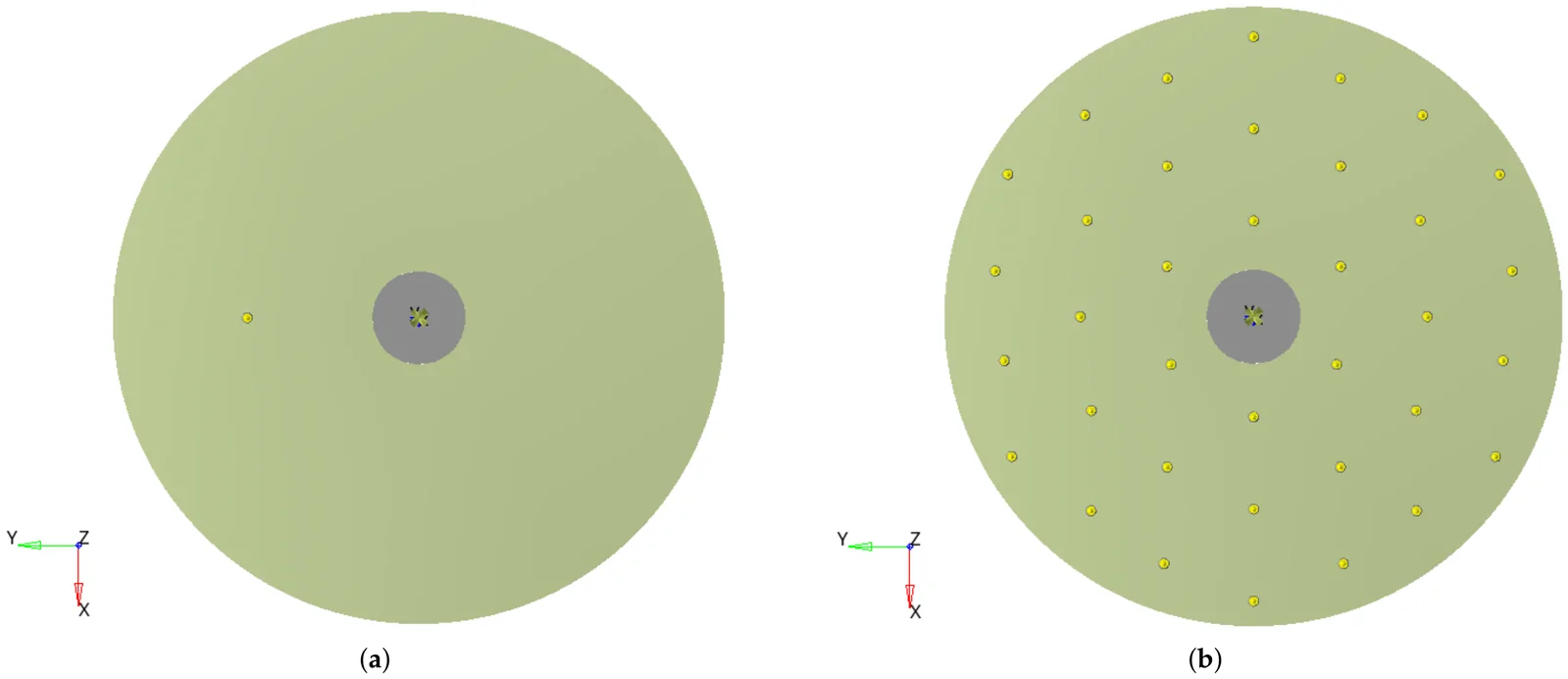
Finite-element models: (**a**) single-actuator model; and (**b**) multi-actuator model.

The FE model uses the properties of the Zerodur-like low-CTE glass ceramic listed in Table 2. The finite-element model is constructed and meshed in Altair HyperMesh 2022 and solved using the companion Altair OptiStruct linear-static solver. The mirror is discretized using four-node, first-order CQUAD4 shell elements, with section properties specified by a PSHELL card. Front-surface nodes provide the optical-surface displacements, whereas active support loads and the central-flexure constraint are applied to the rear surface. Each unit-load case consists of a concentrated 1 N axial force applied at the corresponding support location on the rear surface; positive and negative force values represent push and pull, respectively. In the global model, the central flexure is represented by an equivalent six-degree-of-freedom spring. One end is connected to the mirror’s central attachment region through a distributed-coupling constraint, and the other is attached to a fixed reference point. The equivalent stiffnesses are obtained from a detailed static model by imposing a unit translation or rotation at the flexure–mirror interface and calculating the corresponding reaction force or moment. Because the flexure is approximately symmetric, its cross-coupling stiffnesses are small relative to the principal terms; only the six diagonal stiffnesses are therefore retained in the global optimization model. Their values are $K_{X}$ = 8554 N/mm, $K_{Y}$ = 8568 N/mm, $K_{Z}$ = 234 N/mm, $K_{RX}$ = 1.39 × 10^6^ N mm/rad, $K_{RY}$ = 1.38 × 10^6^ N mm/rad, and $K_{RZ}$ = 5.79 × 10^7^ N mm/rad. This equivalent representation captures the constraints imposed on global rigid-body motion and low-order mirror deformation but is not intended for evaluating local flexure stresses. Figure 7 shows the single- and multi-actuator FE models.

**Figure 8:**
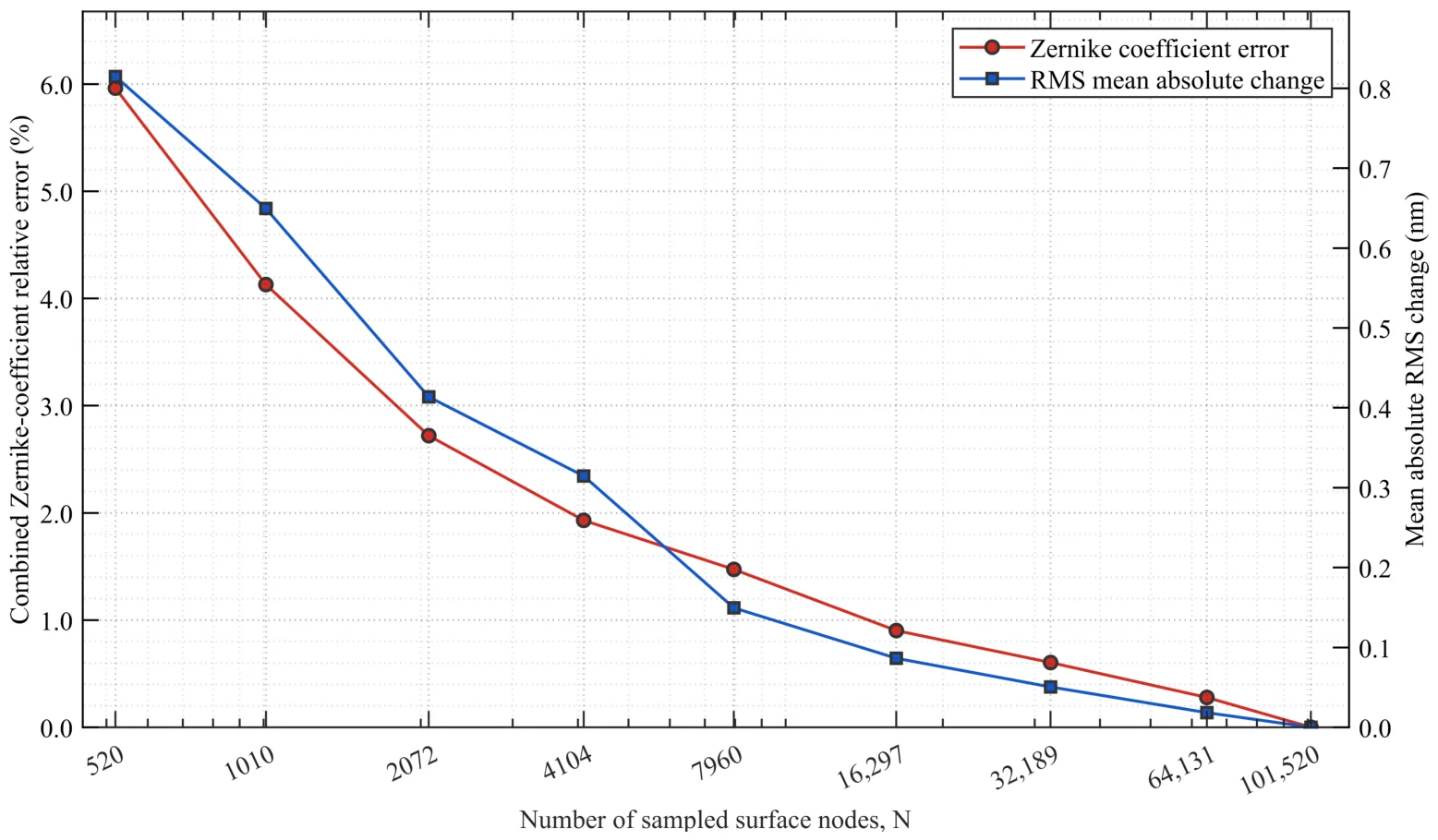
Mesh-convergence histories of the relative Zernike-coefficient error and the mean absolute RMS change with respect to the number of mirror-surface nodes.

A mesh-convergence study is performed while holding the mirror geometry, material properties, element type, boundary conditions, support configuration, and unit-load cases fixed. The tested meshes contain approximately 520, 1010, 2072, 4104, 7960, 16,297, 32,189, 64,131, and 101,520 mirror nodes. At approximately 16,297 nodes, the relative error in the unit-force Zernike coefficients falls below 1%, and the mean absolute change in the mirror RMS falls below 0.1 nm. The solution has therefore entered the converged regime shown in Figure 8, beyond which refinement yields only marginal improvement. To minimize support-to-mesh mapping errors, maintain consistent surrogate-model sampling, and ensure reliable optimization, the refined mesh containing 101,520 mirror nodes and 101,160 elements is adopted as the baseline FE model. Among these nodes, the front-surface nodes within the annular aperture are used to compute the Annular Zernike modal coefficients, with the inner product evaluated as an equally weighted discrete sum over these nodes.

### 5.2. Optical Surface Extraction and Annular Zernike Fitting

**Figure 9:**
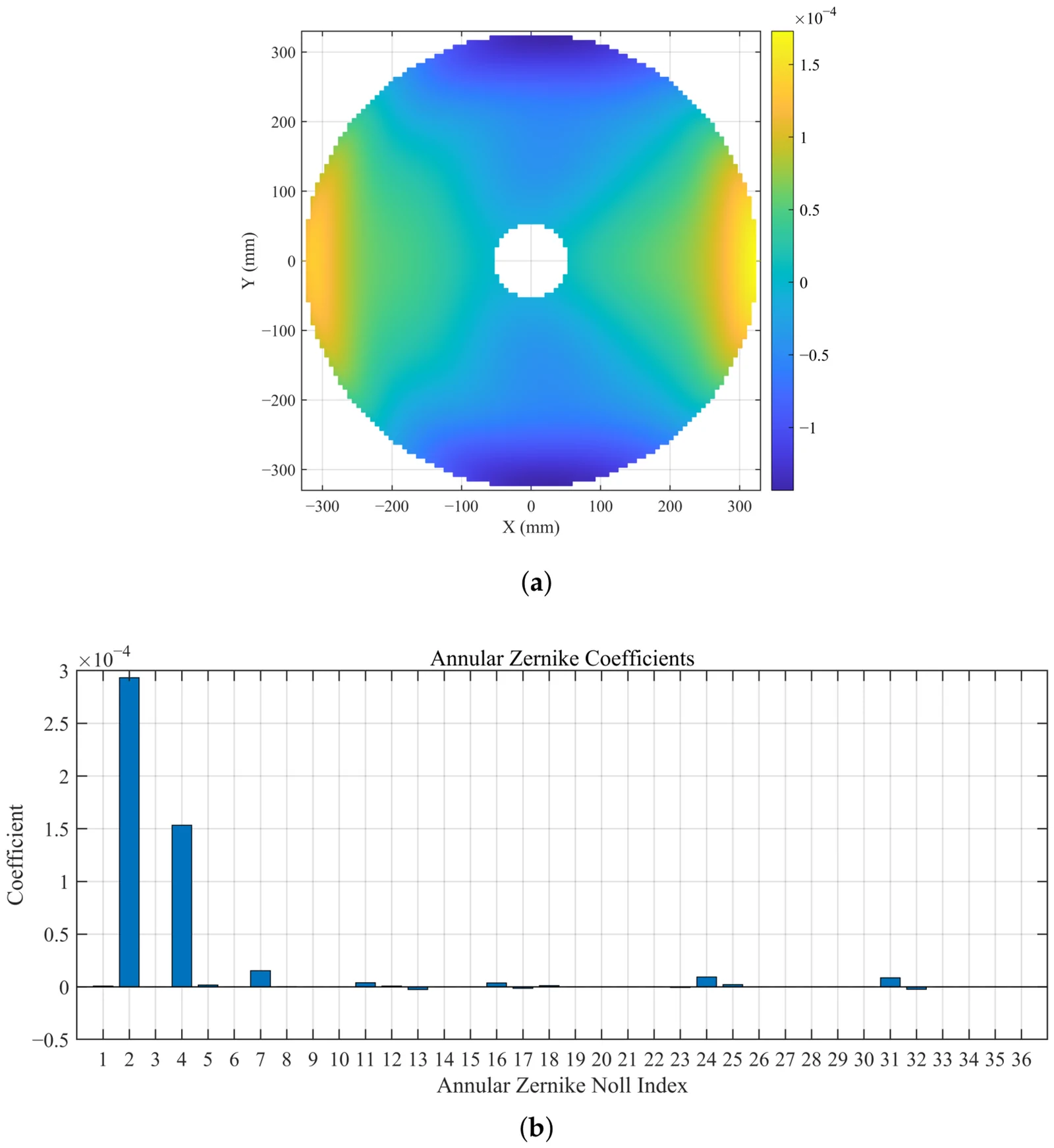
Optical-response extraction and modal decomposition: (**a**) mirror-surface response to a single actuator; and (**b**) the first 36 Annular Zernike coefficients of the response.

To construct the response matrix, the unit-force influence function is evaluated at each candidate support location. A 1 N axial load is applied to a single support pad, and the resulting optical-surface displacement is extracted from the front-surface nodes, as shown in Figure 9a. The displacement field is then decomposed into Annular Zernike coefficients over the aperture. Using the geometric obscuration ratio of 0.1515, the first 36 coefficients are retained to construct the response matrix (Figure 9b).

Because the mirror geometry, material distribution, and constraints are approximately rotationally symmetric about the optical axis, the unit-force response at an arbitrary polar location (R,θ) can be obtained by rotating the reference response at the same radius. Under this rotation, the axisymmetric Annular Zernike coefficients remain unchanged, whereas paired sine and cosine coefficients of the same radial order transform linearly with the rotation angle. The coefficients associated with astigmatism, coma, and trefoil can therefore be recovered from their reference-angle values. Consequently, the polar angle need not be included as an independent surrogate-model input, and the single-actuator response model can be reduced from W(R,θ,h) to W(R,h). This reduction decreases the required number of FE samples and accelerates response-matrix construction.

### 5.3. Kriging Surrogate Model

After rotational reduction, the surrogate-model inputs are the mirror thickness *h* and support radius *R*. Thirteen thicknesses from 1.6 to 4.0 mm are sampled at 0.2 mm intervals, together with 131 radial positions spanning approximately 60–320 mm, yielding 1703 FE samples. At a design point (R,h), the *j*th Annular Zernike coefficient is represented by Equation (9):

(9) $$Z_{j}\left(R,h\right)=\mu_{j}\left(R,h\right)+\eta_{j}\left(R,h\right),\;1\leq j\leq 36,$$

where $\mu_{j}$ is the global trend and $\eta_{j}$ is the local stochastic component. The Gaussian correlation function is defined by Equation (10) [39]:

(10) $$\mathrm{corr}\left[\eta_{j}\left(u\right),\eta_{j}\left(v\right)\right]=\exp\left[-\sum_{k}\theta_{k}{\left|u_{k}-v_{k}\right|}^{2}\right].$$

Here, *u* and *v* are arbitrary sample points, and $\theta_{k}$ is the correlation parameter associated with the *k*th design variable; its magnitude characterizes the response sensitivity in that direction. The 1703 samples are randomly partitioned into training, validation, and test sets containing 70%, 15%, and 15% of the data, respectively. Constant, linear, and quadratic global trends are compared during model selection, together with suitable initial correlation parameters. A quadratic trend with a Gaussian correlation function (regpoly2-corrgauss) is ultimately selected. After model selection, an independent Kriging model is reconstructed for each of the first 36 Annular Zernike coefficients using the combined training and validation samples, which account for 85% of the complete dataset, with the remaining samples retained for independent testing and performance evaluation. This model is subsequently used for the error-metric evaluation and the optimization described below.

**Figure 10:**
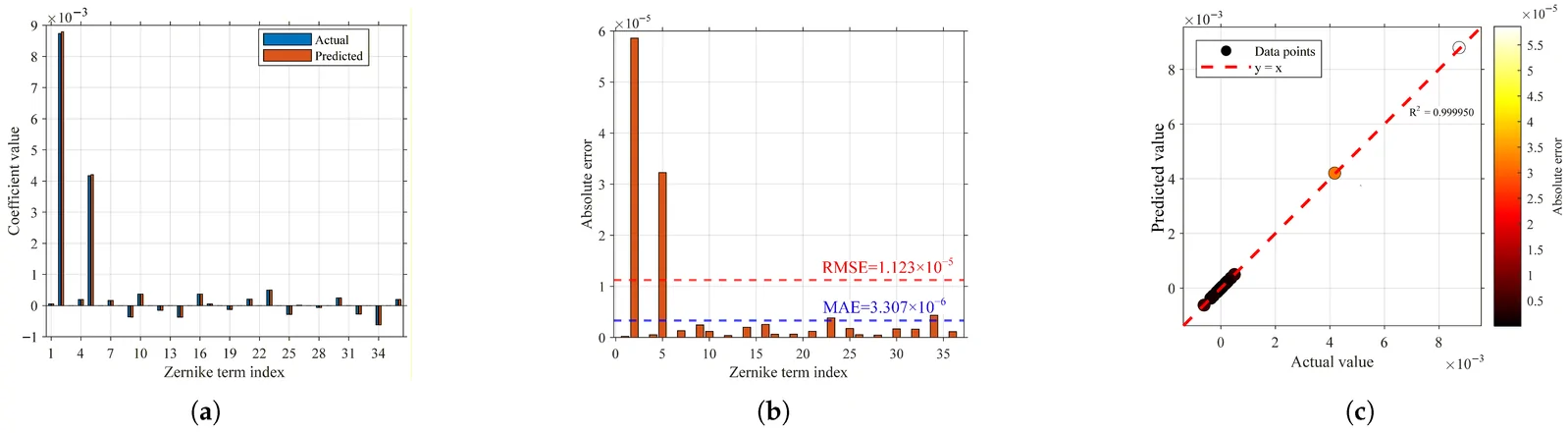
Validation of the Kriging surrogate model: (**a**) comparison of FE and surrogate predictions; (**b**) absolute prediction errors; and (**c**) coefficient of determination.

Surrogate accuracy is assessed on the test set by comparing the Kriging predictions with direct FE results. The evaluation metrics are the mean absolute error (MAE), root mean square error (RMSE), and coefficient of determination *R*^2^. As shown in Figure 10a, the predicted signs and amplitudes of the dominant Annular Zernike coefficients agree closely with the FE results, and their relative errors remain below 1%. Over the test set, the overall RMSE and MAE are 1.123 × 10^−5^ mm and 3.307 × 10^−6^ mm, respectively (Figure 10b), and *R*^2^ = 0.99995 (Figure 10c). Several weak high-order terms exhibit larger relative errors because their true magnitudes are extremely small; nevertheless, their absolute errors remain below 3.502 × 10^−6^ mm and have a negligible effect on the predicted surface response.

**Figure 11:**
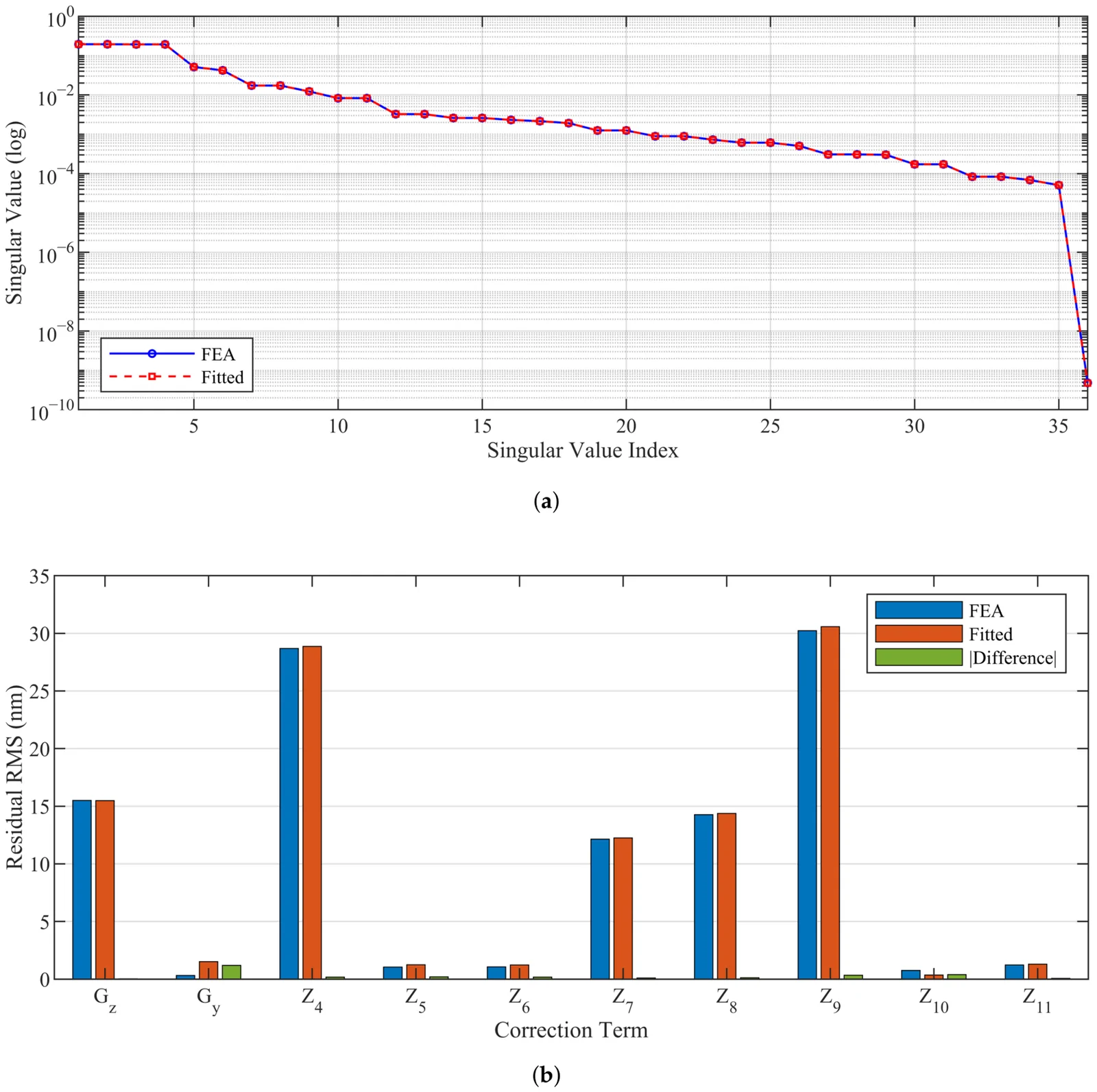
Comparison of the surrogate and direct FE response matrices: (**a**) singular-value spectra; and (**b**) corrected RMS residuals.

The surrogate and direct FE response matrices are also compared. Their leading singular values are nearly coincident (Figure 11a); the maximum relative singular-value error is approximately 0.11%, and the effective rank remains 35. Thus, the Kriging model accurately predicts the individual Annular Zernike coefficients while preserving the modal coverage and numerical characteristics of the complete response matrix. For the 10 target cases in Figure 11b, the mean and maximum absolute differences between the surrogate- and FE-based corrected RMS residuals are 0.2908 nm and 0.9535 nm, respectively. Both are below 1 nm. The surrogate therefore provides sufficient fidelity for influence-function interpolation, response-matrix assembly, controllability analysis, and layout screening during optimization.

## 6. Optimization and Results

### 6.1. Optimization Model

The support layout determines the modal coverage of the response matrix and hence its compatibility with the target space. The VTRI node coordinates are therefore selected as design variables and optimized subject to six-sector rotational symmetry and the prescribed installation-spacing constraint.

#### 6.1.1. Design Variables

The effective layout domain of the annular mirror is defined by Equation (11):

(11) $$\Omega =\{\left(R_{i},\theta_{i}\right)|r_{\min}\leq R_{i}\leq r_{\max},\;0\leq \theta_{i}<\pi /3\}.$$

Here, $r_{\min}$ and $r_{\max}$ are the inner and outer allowable support radii. Owing to six-sector rotational symmetry, only the independent supports within the basic sector, 0° ≤ *θ* < 60°, must be parameterized. The radial and azimuthal coordinates, $R_{i}$ and $\theta_{i}$, of these supports are collected into a single design vector.

For $N_{s}$ independent supports, the design vector is defined by Equation (12):

(12) $$x={\left[R_{1},\theta_{1},R_{2},\theta_{2},\ldots ,R_{N_{s}},\theta_{N_{s}}\right]}^{T}.$$

Here, $N_{s}$ is the number of independent support points in the basic sector.

The full VTRI layout is generated by the rotational replication in Equation (13):

(13) $$P_{\mathrm{vtri}}=\left\{p_{ik}=O\left(k\pi /3\right)p_{i0}|i=1,\ldots ,N_{s},\;k=0,\ldots ,5\right\}.$$

Here, O(·) is the planar rotation matrix, and $p_{i0}$ is the coordinate of the *i*th support in the basic sector.

#### 6.1.2. Constraints

Four classes of constraints are imposed. First, every support must remain within the allowable annulus, whose inner and outer radii are 60 mm and 320 mm, respectively. Second, the Euclidean distance between any two supports must be at least $d_{\min}=50$ mm. Third, the minimum internal angle of every triangular element must be at least $\varphi_{\min}=40^{{}^{\circ}}$ to maintain local support efficiency and mesh quality. Fourth, for each target surface, the infinity norm of the least-squares correction-force vector must not exceed the actuator-force limit. The specified value, $f_{\max}=0.5$ N, is the maximum incremental active correction force relative to the preloaded operating point; it is not the total static support force carried by an actuator. Collectively, these constraints are expressed as $60\leq R_{i}\leq 320$ mm, $∥p_{i}-p_{j}{∥}_{2}\geq 50$ mm, $\varphi_{q,m}\geq 40^{\circ }$, and the force constraint in Equation (14):

(14) $$∥f^{\left(k\right)}{∥}_{\infty }\leq f_{\max},\;f_{\max}=0.5\;N,\;k=1,\ldots ,10.$$

A quadratic penalty $P_{\mathrm{geo}}\left(x\right)$ is added to the objective function when a candidate layout violates a boundary, spacing, or internal-angle constraint, thereby enforcing manufacturability and alignment feasibility.

#### 6.1.3. Objective Function

The optimization targets comprise the two gravity cases and the low-order Annular Zernike modes *Z*_4_–*Z*_11_. For the *k*th target, the actuator-force vector is determined by minimizing the difference between the target Annular Zernike coefficients and the response-matrix output.

The unconstrained least-squares solution $f_{k}\left(x\right)=H{\left(x\right)}^{+}a_{k}$ is evaluated first. If $∥f_{k}{\left(x\right)∥}_{\infty }\leq f_{\max}$, it is accepted directly. Otherwise, a damped least-squares solution is used, and the damping parameter $\lambda_{k}$ is determined by a one-dimensional search such that $∥f_{k}{\left(x\right)∥}_{\infty }$ approaches $f_{\max}$. This regularized solution minimizes the fitting residual at the force-limit boundary and is expressed by Equation (15):

(15) $$f_{k}\left(x\right)=\left\{\begin{array}{cc} H{\left(x\right)}^{+}a_{k},\; & {∥H{\left(x\right)}^{+}a_{k}∥}_{\infty }\leq f_{\max},\\ {\left[H{\left(x\right)}^{T}H\left(x\right)+\lambda_{k}I\right]}^{-1}H{\left(x\right)}^{T}a_{k},\; & {∥H{\left(x\right)}^{+}a_{k}∥}_{\infty }>f_{\max}. \end{array}\right.$$

The corrected RMS residual is used as the surface-error metric. Compared with the peak-to-valley (PV) value, RMS more robustly characterizes the error over the entire clear aperture and is therefore better suited to active support layout optimization. Substituting $f_{k}\left(x\right)$ gives Equation (16):

(16) $$R_{k}\left(x\right)=\mathrm{RMS}\left(a_{k}-H\left(x\right)f_{k}\left(x\right)\right).$$

Because the target surfaces have different amplitudes and residual requirements, a weighted sum converts the multitarget problem into a scalar objective. Axial and radial gravity are deterministic operational loads and are assigned greater weight than the low-order *Z*_4_–*Z*_11_ aberrations. The resulting weight vector is ${\left[2,2,1,1,1,1,1,1,1,1\right]}^{T}$.

The weighted residual objective is defined by Equation (17):

(17) $$J_{\mathrm{rms}}\left(x\right)=\sum_{k\in K}w_{k}{\left(\frac{R_{k}\left(x\right)}{R_{\mathrm{req},k}}\right)}^{2},$$

where K={1,2,…,10} is the set of target cases, $w_{k}$ is the corresponding weight, and $R_{\mathrm{req},k}$ is the RMS residual requirement. The complete objective combines the weighted residual and geometric penalty terms:

(18) $$J\left(x\right)=J_{\mathrm{rms}}\left(x\right)+P_{\mathrm{geo}}\left(x\right).$$

Equation (18) defines the VTRI optimization problem under six-sector symmetry while accounting jointly for gravity correction, low-order aberration correction, actuator-force limits, and geometric feasibility.

### 6.2. Optimization Procedure and 36-Point VTRI Result

CMA-ES is selected for the global search because it is well suited to nonlinear, nonconvex, derivative-free optimization of expensive black-box functions [40]. Through repeated sampling, selection, and covariance-matrix updates, the algorithm learns correlations among the design variables and adapts its search distribution. The population size is scaled with the design dimension. Each candidate is projected onto the allowable domain and checked for spacing violations before its surrogate response matrix, correction residual, and penalty terms are evaluated.

The optimization proceeds in two stages. CMA-ES first identifies promising regions of the design space, after which the best candidate is refined locally to reduce the residual further while preserving feasibility. The 36-point VTRI layout has 12 independent variables in the basic sector, and a population size of 18 is used. The maximum number of evolutionary generations is set to 300, corresponding to a maximum of 18 × 300 = 5400 function evaluations for the CMA-ES global-search stage. This moderately enlarged population improves design-space coverage within each generation. Each variable is normalized by its allowable range, and its initial standard deviation is set to one-sixth of that range. This choice reflects the combined restrictions imposed by the 50 mm minimum spacing, the 40° minimum triangle angle, and the sector boundaries. A larger initial standard deviation would generate many infeasible layouts, whereas a smaller value would restrict early global exploration. CMA-ES terminates when the objective improves by less than 10^−3^ over 50 consecutive valid generations. In the second stage, the best solution obtained by CMA-ES serves as the initial point for a local pattern-search refinement. Fourteen geometrically decreasing step sizes, ranging from 0.12 to 2 × 10^−5^, are applied sequentially; at each step size, the 12 design variables are perturbed one at a time in both the positive and negative directions from the current best point, generating 12 × 2 = 24 candidate points per step size, and any perturbation that reduces the objective function is accepted as the new incumbent. The search proceeds through all 14 step sizes in decreasing order, providing a bounded, finite-termination local refinement of the CMA-ES solution, with a maximum of 14 × 12 × 2 = 336 additional function evaluations for this stage. The final support layout is then selected, from among the candidate solutions satisfying the support-radius, minimum-spacing, and minimum-internal-angle constraints, according to the minimum value of the composite objective function J(x) obtained after optimization.

**Figure 12:**
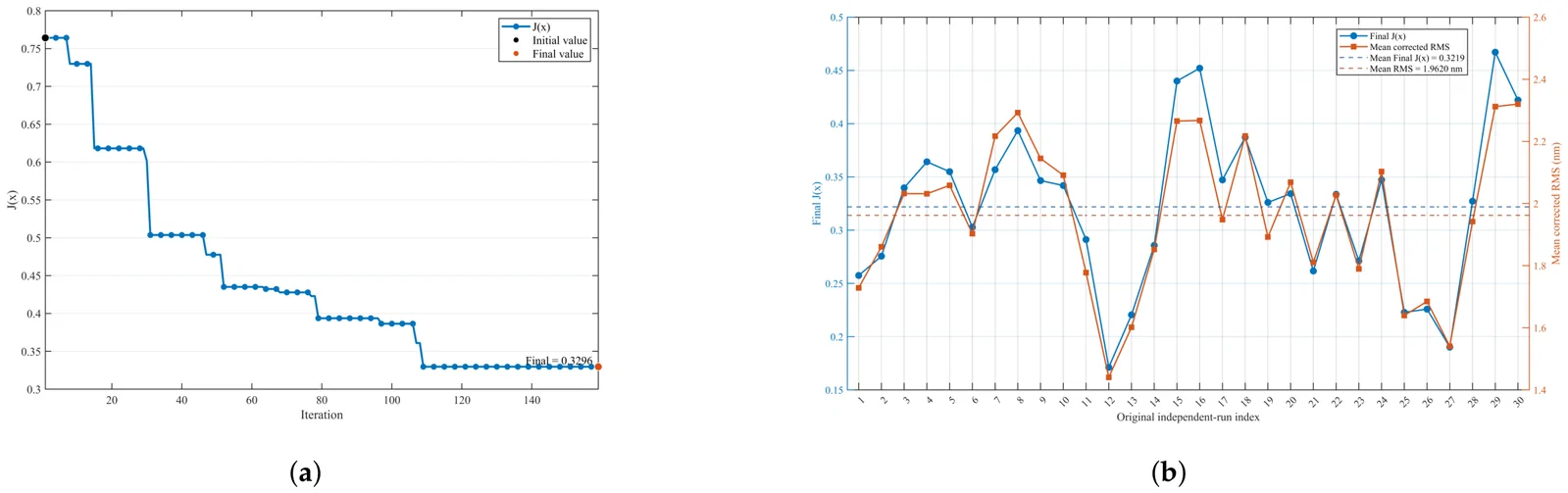
CMA-ES convergence and repeatability for the VTRI layout: (**a**) objective-function history; and (**b**) final objective-function values and mean corrected RMS residuals from 30 independent runs.

Algorithm repeatability is assessed using 30 independent CMA-ES runs. At convergence, the objective-function value is 0.3219 ± 0.075, corresponding to a coefficient of variation of 23.28%. The mean corrected RMS residual is 1.9620 ± 0.2430 nm, with a coefficient of variation of 12.39%. Figure 12a shows that the objective decreases substantially within a limited number of generations. The 30-run statistics in Figure 12b show comparatively low dispersion in the mean corrected RMS residual, indicating good repeatability.

**Figure 13:**
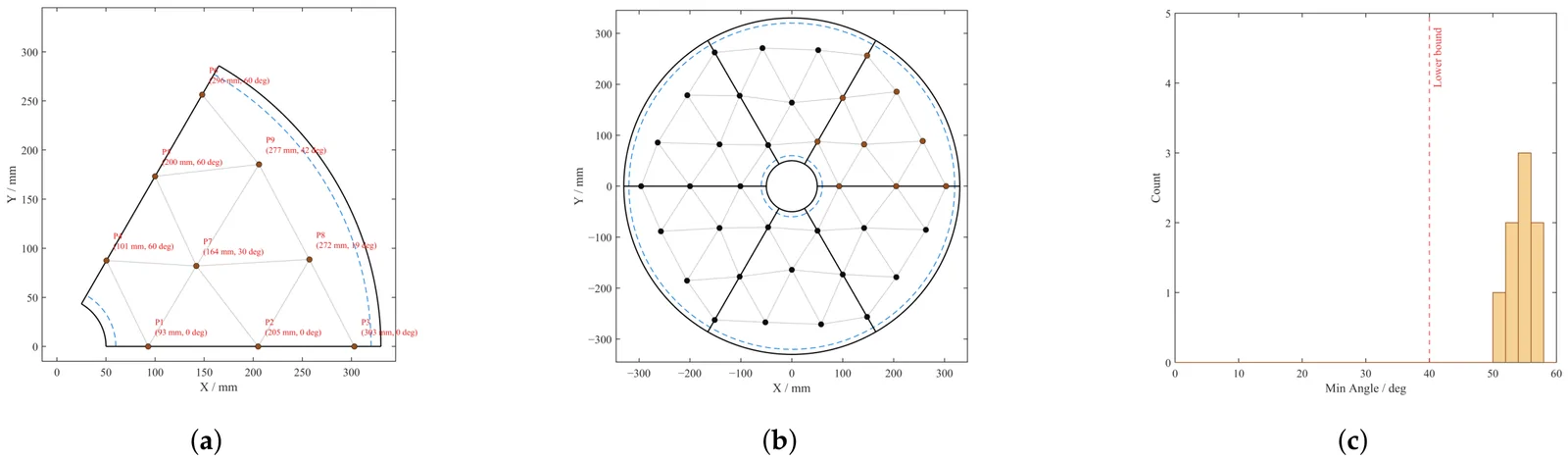
Optimized 36-point VTRI layout: (**a**) optimized node coordinates in the basic sector; (**b**) six-fold replicated triangular network; and (**c**) distribution of triangle internal angles.

The surrogate model is trained once offline and evaluated repeatedly during optimization. All variables remain within the FE sampling ranges 60 ≤ *R* ≤ 320 mm and 1.6 ≤ *h* ≤ 4.0 mm. Within the basic 60° sector, the nodes can move locally but must preserve the triangular-grid topology and satisfy the spacing and boundary constraints. This parameterization balances local support efficiency with engineering feasibility. Figure 13a–c shows the coordinates, replicated mesh, and internal-angle distribution of the optimized 36-point VTRI layout. (Unless otherwise specified, all results presented in this study are evaluated at the nominal reference mirror thickness of *h* = 2.0 mm.) The solution satisfies the boundary, spacing, mesh-quality, and actuator-force constraints while retaining six-fold symmetry. It also provides adequate clearance for the actuators, support pads, and flexural connectors and establishes a regular geometry for fabrication and alignment. Local node adjustment can therefore improve target-space coverage without sacrificing engineering symmetry.

Correction performance is evaluated for three groups of targets: surface-figure errors induced by axial and radial gravity, the individual low-order Annular Zernike modes, and a combined aberration formed by superposing 40% of the prescribed amplitude of each *Z*_4_–*Z*_11_ component. Table 4 summarizes the target and corrected RMS values.

**Figure 14:**
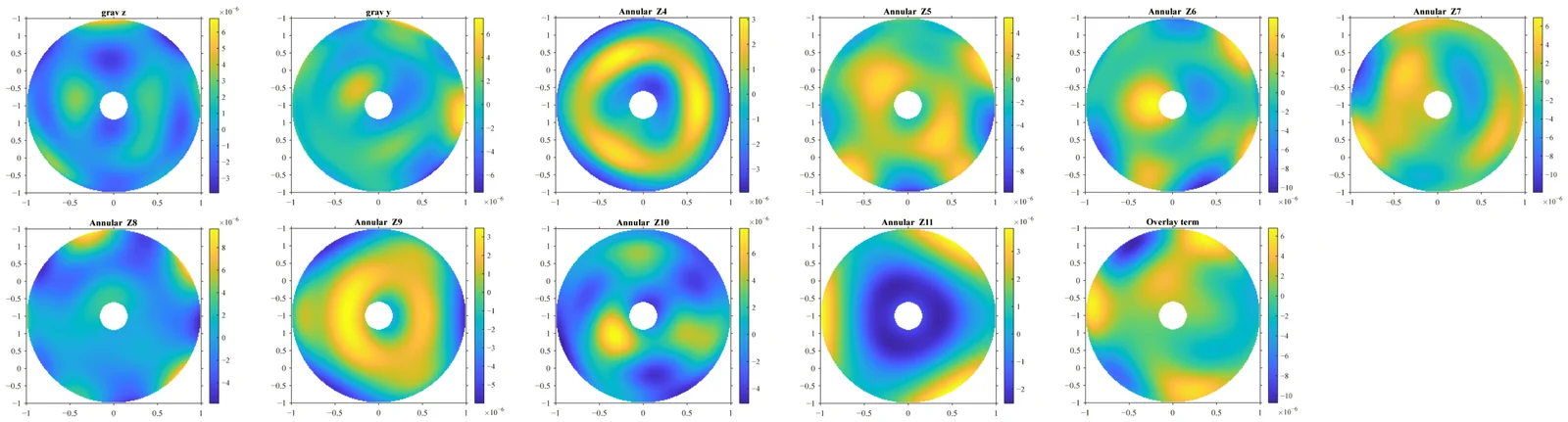
Residual surface maps after active correction of the gravity, individual aberration, and combined-aberration targets.

As reported in Table 4, the corrected RMS residuals for axial gravity, radial gravity, the individual *Z*_4_–*Z*_11_ modes, and the combined aberration all remain well below the 12.66 nm requirement. The correction ratios exceed 97.8% for all *Z*_4_–*Z*_11_ modes and are approximately 97.9% and 79.2% for axial and radial gravity, respectively. For the combined aberration, the RMS is reduced from 245 nm to 3.06 nm. Figure 14 compares the corresponding residual surface distributions.

The optimized layout also exhibits a clear radial distribution of correction roles. Placing the outer supports too close to the edge can introduce local stiffness discontinuities and high-spatial-frequency print-through. Supports at intermediate radii balance the correction of gravity, astigmatism, coma, and spherical aberration, whereas the inner supports primarily affect spherical aberration, trefoil, and the response near the central aperture. This radial complementarity enables VTRI to maintain a low composite residual under the actuator-force constraint.

### 6.3. Analysis of Support-Layout Characteristics

#### 6.3.1. Effect of Support Count

Increasing the support count generally improves spatial sampling and modal coverage, but support count alone does not determine correction performance. For a fixed number of actuators, different topologies can produce substantially different response-matrix condition numbers, tail singular values, and force demands. Support count must therefore be considered together with topology, radial stratification, azimuthal symmetry, and force distribution.

The VTRI and reference layouts are compared at support counts of 18, 21, 24, 27, 30, 33, and 36. Because a square lattice is severely truncated at several of these counts, SQ layouts with 20, 24, 28, 32, and 36 supports are used instead. TRI, SQ, and HEX are parameterized by the lattice spacing *S* and global rotation; their underlying topologies remain fixed during optimization. ANN uses several concentric rings, with more supports assigned to the outer rings to maintain comparable sampling density across radial zones. For each support count, the numbers of rings and supports per ring are prescribed, and the ring radii and relative phase offsets are optimized. FIB uses area-uniform radial sampling and golden-angle azimuthal recursion. Its global rotation, spiral-angle correction, radial offset, and radial-distribution exponent are optimized to improve coverage of the target aberration space.

**Figure 15:**
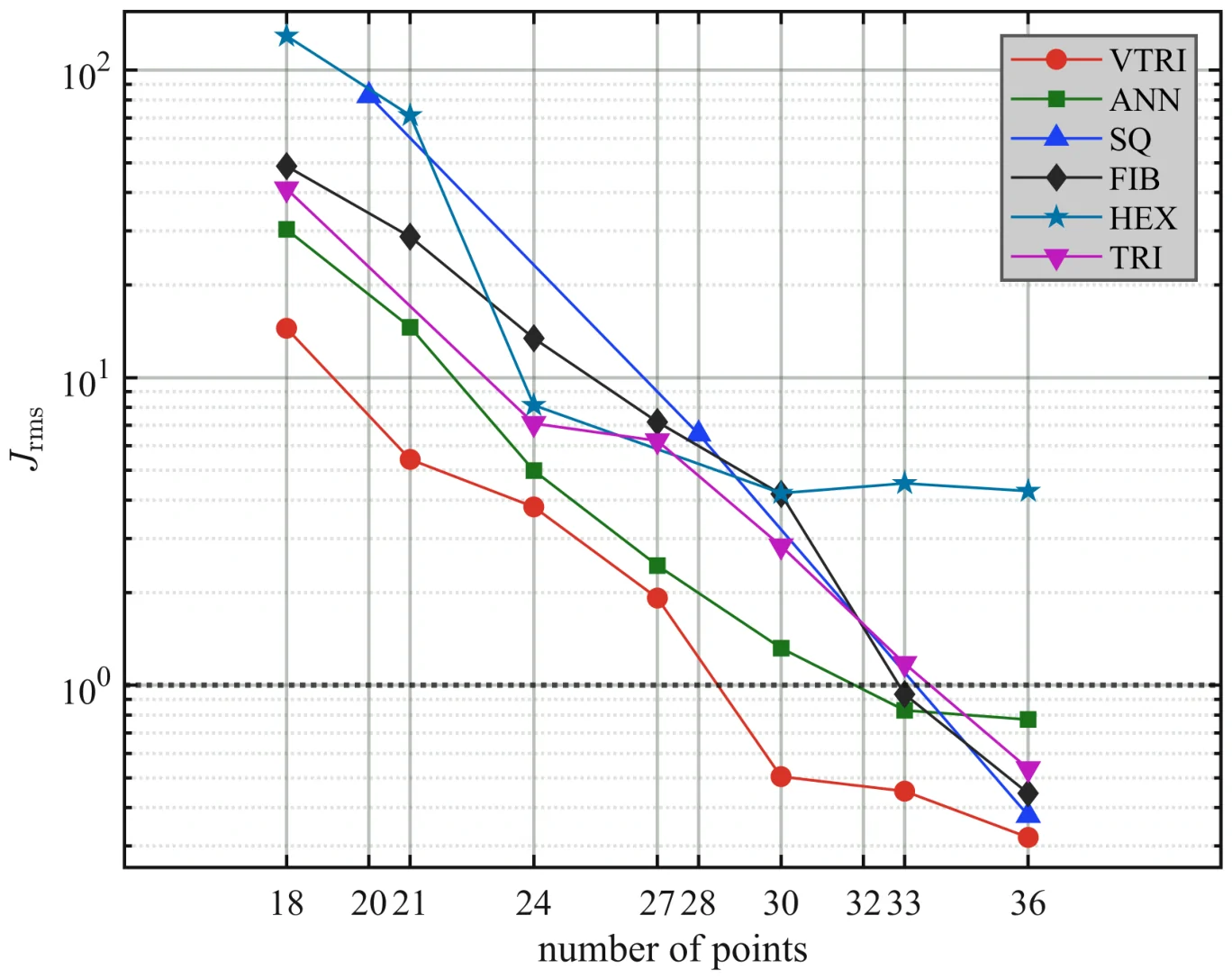
The weighted RMS residual of the optimized layouts at different support counts.

Figure 15 compares the weighted RMS residual across the support-count schemes. The weighted RMS residual generally decreases as the number of supports increases, although the rate of improvement depends on topology. VTRI provides the best overall performance among the parameterized layouts. At 36 supports, it satisfies the low-order aberration requirement while retaining adequate actuator-force margin and installation clearance; the 36-point VTRI configuration is therefore selected for further analysis.

#### 6.3.2. Comparison of Optimized Layouts with Different Parameterizations

**Figure 16:**
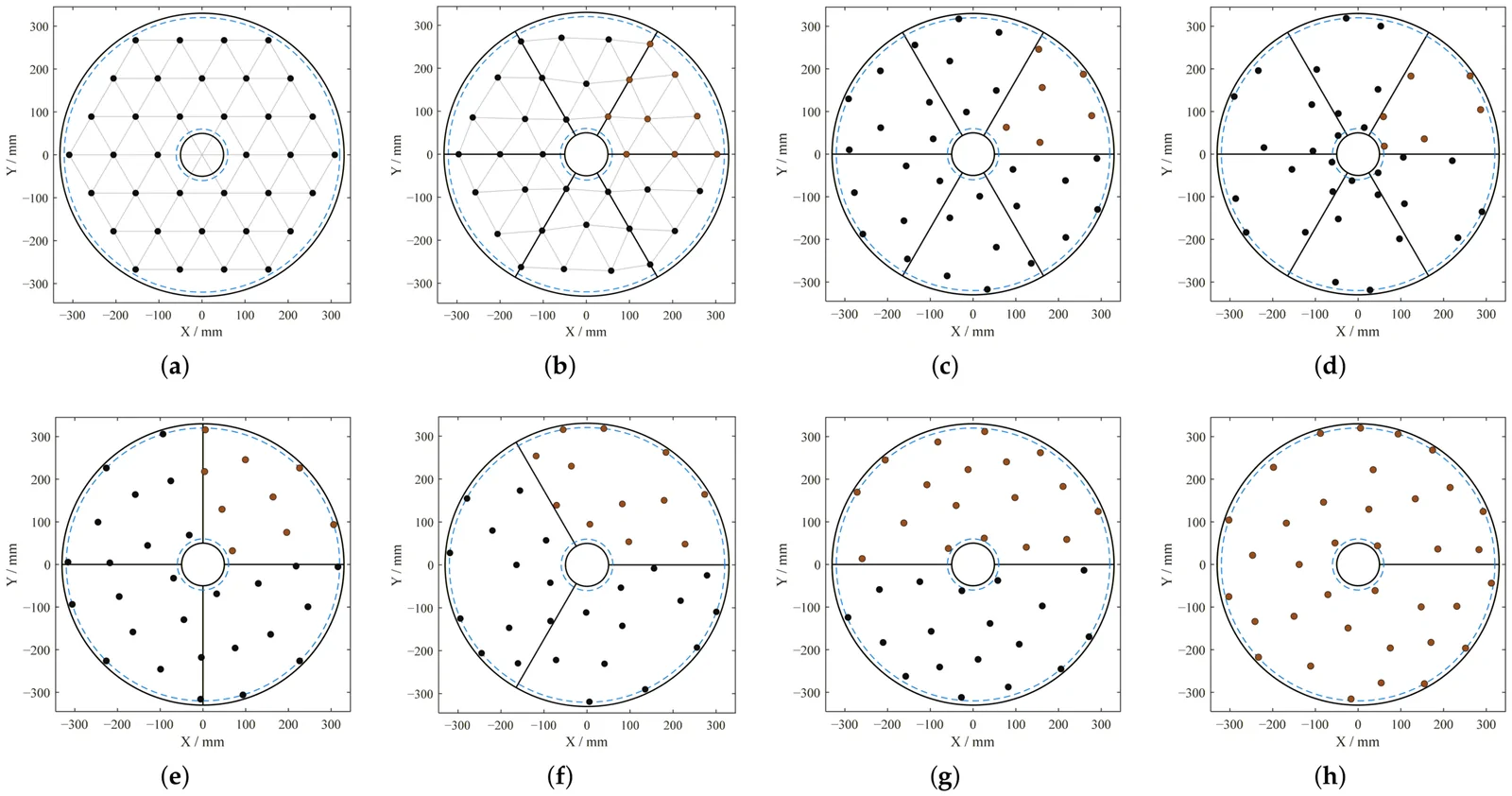
Optimized support layouts with different symmetry orders and numbers of design variables: (**a**) TRI; (**b**) VTRI; (**c**) 6-array-80; (**d**) 6-array-50; (**e**) 4-array-80; (**f**) 3-array-80; (**g**) 2-array-80; and (**h**) free-80.

To isolate the effects of parameterization and symmetry, VTRI is compared with alternative 36-point layouts using the correction targets in Table 1 and the CMA-ES computational budget specified in Section 6.2. Seven layout families with different symmetry orders and numbers of design variables are considered. TRI is a rigid triangular lattice with six-fold rotational symmetry and only two variables: lattice scale and global rotation. VTRI also has six-fold symmetry, but the nodes in its basic sector can move locally while satisfying the minimum-angle and triangular-connectivity constraints, resulting in 12 variables. The 6-array layout has the same six-fold symmetry and 12 variables as VTRI, but its sector nodes move independently without connectivity or mesh-quality constraints. The 4-array, 3-array, and 2-array layouts have four-, three-, and two-fold rotational symmetry and contain 18, 24, and 36 variables, respectively. In the free layout, all 36 supports are optimized independently without rotational symmetry, giving 72 variables. Every layout is subject to a minimum support-spacing constraint. For the 6-array, 4-array, 3-array, 2-array, and free layouts, minimum spacings of both 50 and 80 mm are evaluated, with the latter introduced to promote greater spatial uniformity. Table 5 summarizes the optimization results, and Figure 16 shows the representative optimized layouts.

VTRI has more design variables than the baseline TRI layout and consequently achieves a lower correction residual. The 6-array-50 layout has the same 12 variables and 50 mm minimum-spacing constraint as VTRI. Its objective-function value of 0.28 and mean RMS residual of 1.60 nm are lower than the corresponding VTRI values of 0.34 and 2.15 nm. However, Figure 16a–d shows that the absence of minimum-angle and triangular-connectivity constraints makes the optimized 6-array-50 node distribution less uniform and therefore less suitable for engineering implementation. Increasing its minimum spacing to 80 mm improves spatial uniformity but increases the objective-function value to 0.38 and the mean RMS residual to 2.20 nm. The triangular network in VTRI thus provides geometric regularization that balances layout uniformity against correction residual.

From the 12-variable 6-array layout to the 72-variable free layout, the design dimension increases progressively as symmetry is relaxed. The mean RMS residual, however, does not improve monotonically with the size of the search space and generally deteriorates from 1.60 nm among the 50 mm cases. Under a fixed computational budget, increasing the design dimension raises the population and iteration requirements of CMA-ES and makes its covariance matrix more difficult to estimate accurately. The search may therefore converge prematurely to a local optimum, illustrating the practical effect of dimensionality. Moreover, as shown in Figure 16e–h, satisfying the minimum-spacing constraint alone does not prevent irregular distributions or local voids, both of which reduce engineering applicability.

### 6.4. Finite-Element Comparison of Support Layouts

**Figure 17:**
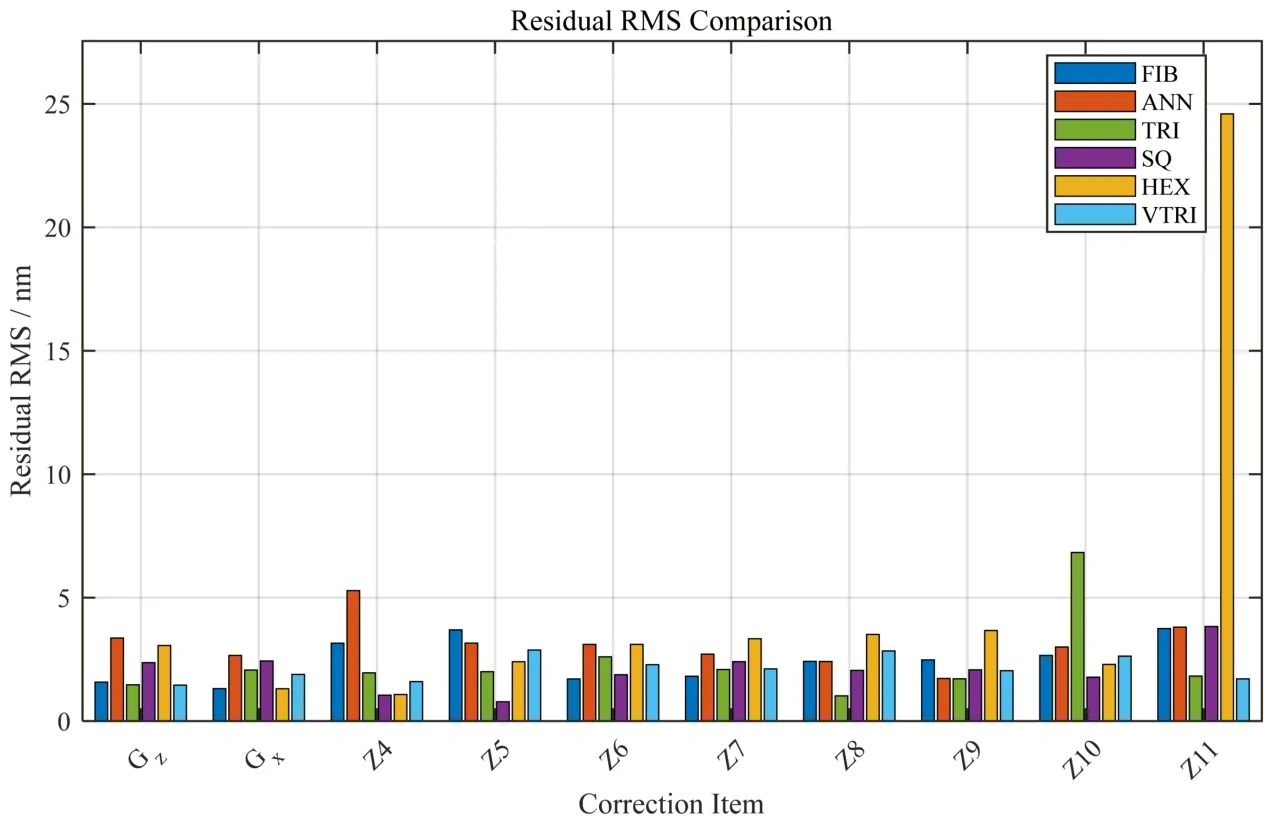
Corrected RMS residuals for the VTRI, ANN, TRI, FIB, HEX, and SQ layouts.

The coordinates of each optimized 36-point layout are reintroduced into the FE model, and all six layouts are evaluated at the same support count. This comparison isolates the effect of topology on correction performance and actuator-force distribution. Figure 17 compares the corrected RMS residuals for low-order, mid-order, and more complex target surfaces. Across the two gravity cases and the Annular Zernike modes *Z*_4_–*Z*_11_, robustness across targets is more important than achieving the minimum residual in an isolated case. VTRI maintains low residuals for most targets and provides the lowest worst-case residual. SQ approaches VTRI for some metrics but exhibits stronger directional bias, whereas ANN provides insufficient coverage of nonaxisymmetric and higher-order modes.

**Figure 18:**
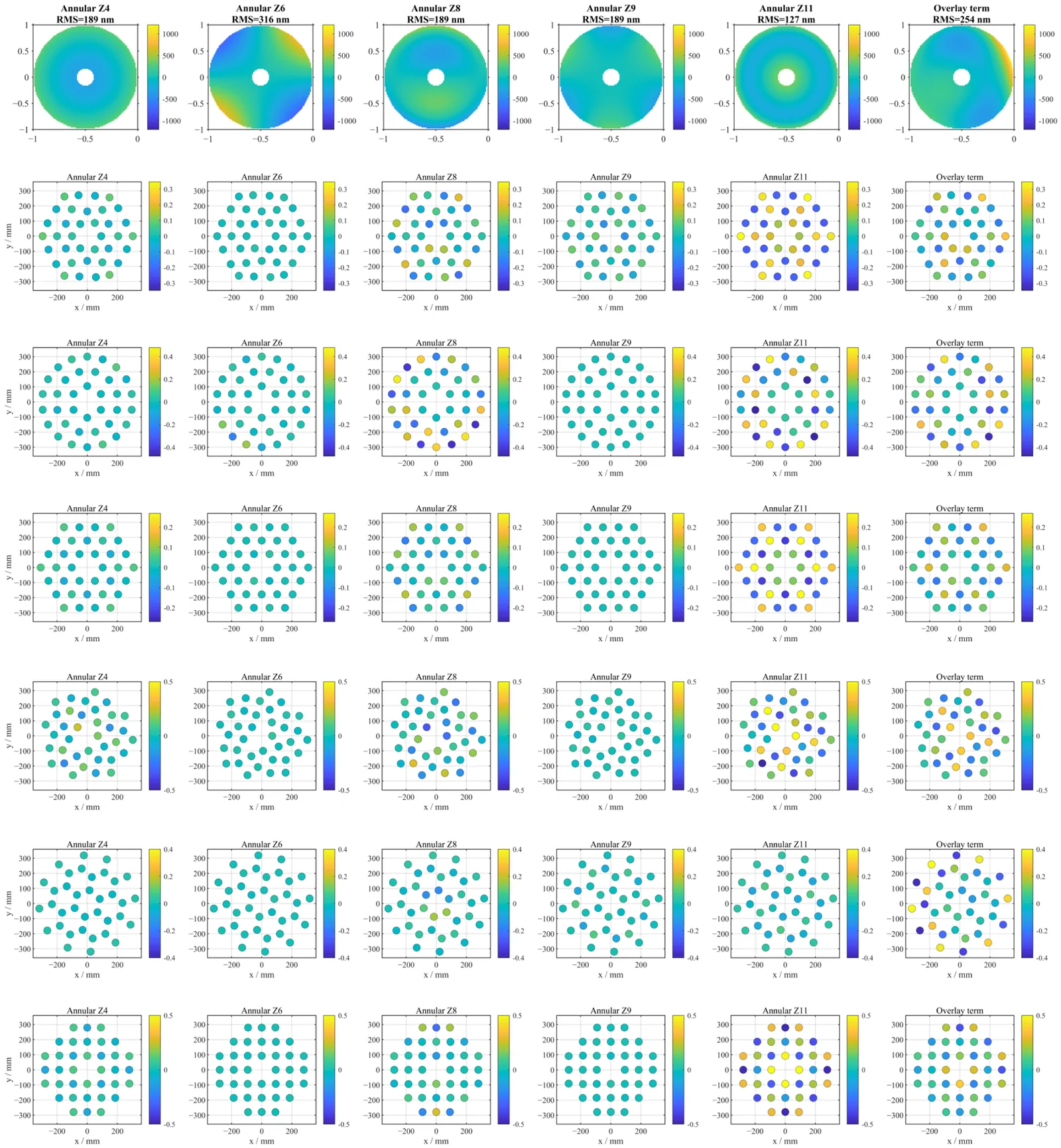
Correction -force distributions for representative targets. The columns correspond to *Z*_4_, *Z*_6_, *Z*_8_, *Z*_9_, *Z*_11_, and the combined-aberration term. From top to bottom, the rows show the target surfaces and the force distributions for VTRI, ANN, TRI, FIB, HEX, and SQ.

The spatial distribution of actuator forces provides further insight into the differences among the layouts. Figure 18 compares the force patterns for representative targets. VTRI distributes the correction effort across the inner, intermediate, and outer radial zones, producing relatively uniform coverage over the aperture. In ANN, the forces remain concentrated on the prescribed concentric rings. TRI, HEX, and SQ exhibit directional patterns associated with their regular lattices, whereas FIB provides broader but less structured coverage. Force magnitude also depends on the target; *Z*_9_ and the combined-aberration term require comparatively large forces. These results demonstrate that support topology governs not only the attainable residual but also the spatial allocation of control effort. The six-fold symmetry of VTRI yields a comparatively balanced force distribution in both the radial and azimuthal directions.

Table 6 summarizes the correction-performance metrics for the six layouts. VTRI yields the lowest composite objective-function value (0.34), a mean RMS residual of 2.15 nm, and an effective rank of 35. The corresponding mean RMS residuals of TRI, SQ, and FIB are 2.36, 2.27, and 2.35 nm, respectively. Although SQ and FIB also attain an effective rank of 35, both their objective values and mean residuals exceed those of VTRI, demonstrating that spatial support distribution affects correction accuracy even at comparable modal ranks. HEX has an effective rank of 31 and the largest mean RMS residual, 4.83 nm. ANN has the lowest effective rank (27) and a mean RMS residual of 3.12 nm. Despite its relatively low condition number, the rank deficiency of ANN leaves several target directions weakly controllable. The mean projection residual measures the average relative magnitude of the component of each *Z*_4_–*Z*_36_ target that lies outside the theoretically controllable subspace. Its near-zero values for VTRI, SQ, and FIB indicate that these 36-point layouts can theoretically span nearly all target modes over *Z*_4_–*Z*_36_. The mean regularization residual measures the average relative difference between each target and the corresponding force-limited, regularized correction response. VTRI has the lowest value (0.563), indicating strong fitting capability while limiting amplification in weak-response directions and avoiding excessive control forces. Collectively, the objective value, RMS residual, effective rank, and subspace metrics show that VTRI provides the best balance of correction accuracy and modal coverage under the constraints considered.

### 6.5. Monte Carlo Analysis of Installation Errors

A Monte Carlo analysis is performed to quantify the sensitivity of the optimized 36-point VTRI layout to installation errors. In practice, the supports are positioned using high-precision fixtures and then aligned through repeated measurement and adjustment relative to an installation datum. Systematic fixture errors are therefore assumed to be negligible compared with the residual pointwise deviations introduced during independent assembly operations, such as bonding and shim adjustment. Because these operations do not share a common error source, the support-position deviations are modeled as mutually independent random variables [28]. Engineering tolerances indicate a random installation deviation of approximately ±1 mm. Accordingly, each support-point perturbation is uniformly sampled within a circular region of radius 1 mm. This model provides a conservative representation of the specified tolerance band rather than a Gaussian error model. During the Monte Carlo analysis, any perturbed layout violating the radial boundary constraint or minimum support-spacing constraint is considered infeasible. In such cases, the layout constraints should be further adjusted, and the support layout should be re-optimized before performing the tolerance analysis.

**Figure 19:**
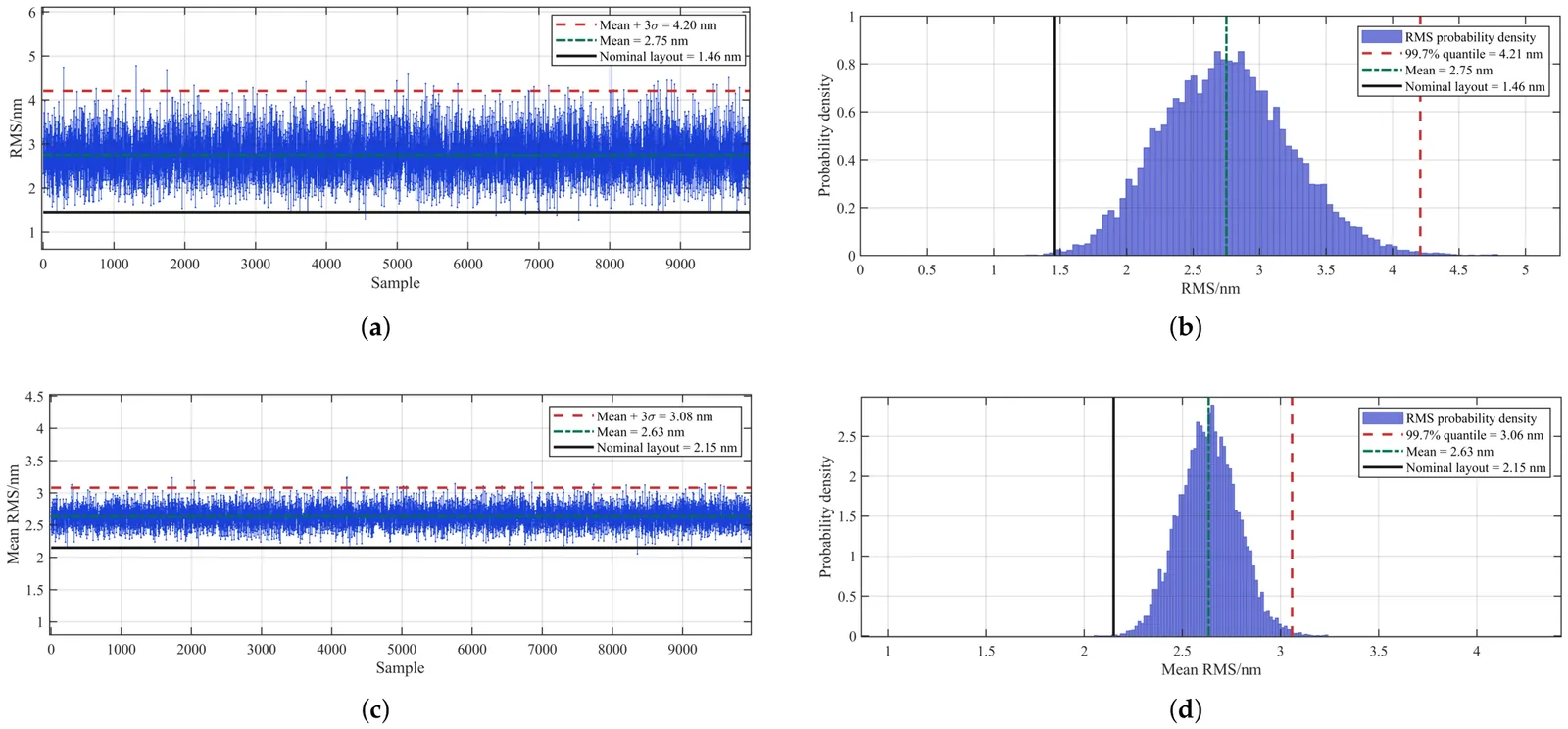
Monte Carlo analysis for ±1 mm support-position errors: (**a**) corrected RMS residuals under axial gravity; (**b**) probability-density distribution for axial gravity; (**c**) mean RMS residuals averaged over the ten target conditions; and (**d**) probability-density distribution of the mean RMS residuals.

The nominal optimized layout serves as the baseline, and 10,000 independently perturbed layouts are generated. Among these 10,000 realizations, none violated the radial boundary constraint or the minimum support-spacing constraint, confirming that the optimized VTRI layout retains sufficient geometric margin under the prescribed ±1 mm installation tolerance. For each realization, the corrected RMS residual under the axial-gravity condition is recalculated to evaluate the influence of support-position errors under a single operating condition. As shown in Figure 19a,b, the installation errors increase the RMS residual under Case 1. The nominal-layout RMS residual is 1.46 nm, whereas the perturbed layouts exhibit a mean RMS residual of 2.75 nm, with a standard deviation of 0.48 nm and a 99.7th-percentile value of 4.21 nm.

To further evaluate the overall correction robustness, the composite corrected RMS residual is calculated as the average RMS residual over the ten target conditions. As shown in Figure 19c,d, the nominal-layout composite RMS residual is 2.15 nm. Under mutually independent ±1 mm support-position errors, the mean composite RMS residual increases to 2.63 nm, with a standard deviation of 0.15 nm and a 99.7th-percentile value of 3.06 nm. These results demonstrate that the optimized VTRI layout maintains stable correction performance and remains robust against independent ±1 mm support-position errors.

### 6.6. Effects of Actuator-Force Limit and Mirror Thickness

**Figure 20:**
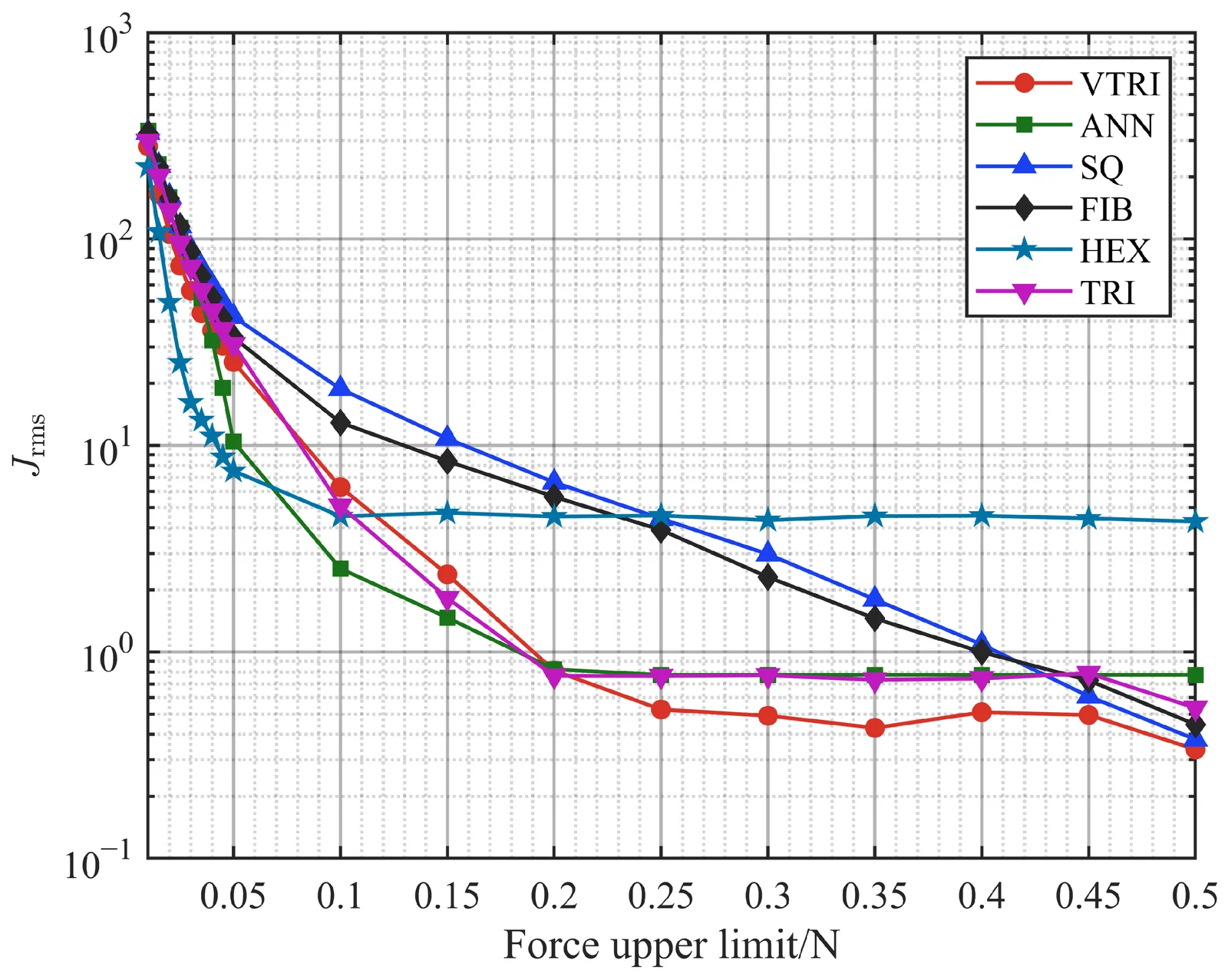
The weighted RMS residual of the six layouts as functions of the incremental actuator-force limit.

The sensitivity to the incremental actuator-force limit is evaluated for all six layouts. Figure 20 shows the residual as the maximum correction-force magnitude is varied for a 2 mm-thick mirror with a force resolution of 0.001 N. Increasing the limit toward 0.5 N substantially reduces the residuals of most layouts, confirming that actuator saturation constrains correction performance at low force limits. VTRI yields the lowest residual at the specified upper limit, followed closely by SQ, FIB, and TRI. The ANN and HEX curves plateau before 0.5 N, indicating that their performance is limited primarily by topology rather than available actuator force. Although the FIB and SQ residuals continue to decrease with increasing force limit, both remain above that of VTRI at 0.5 N. VTRI also provides the lowest residual throughout the 0.2–0.5 N range. Thus, under the 0.5 N incremental force limit adopted in this study, VTRI provides the best final correction performance.

**Figure 21:**
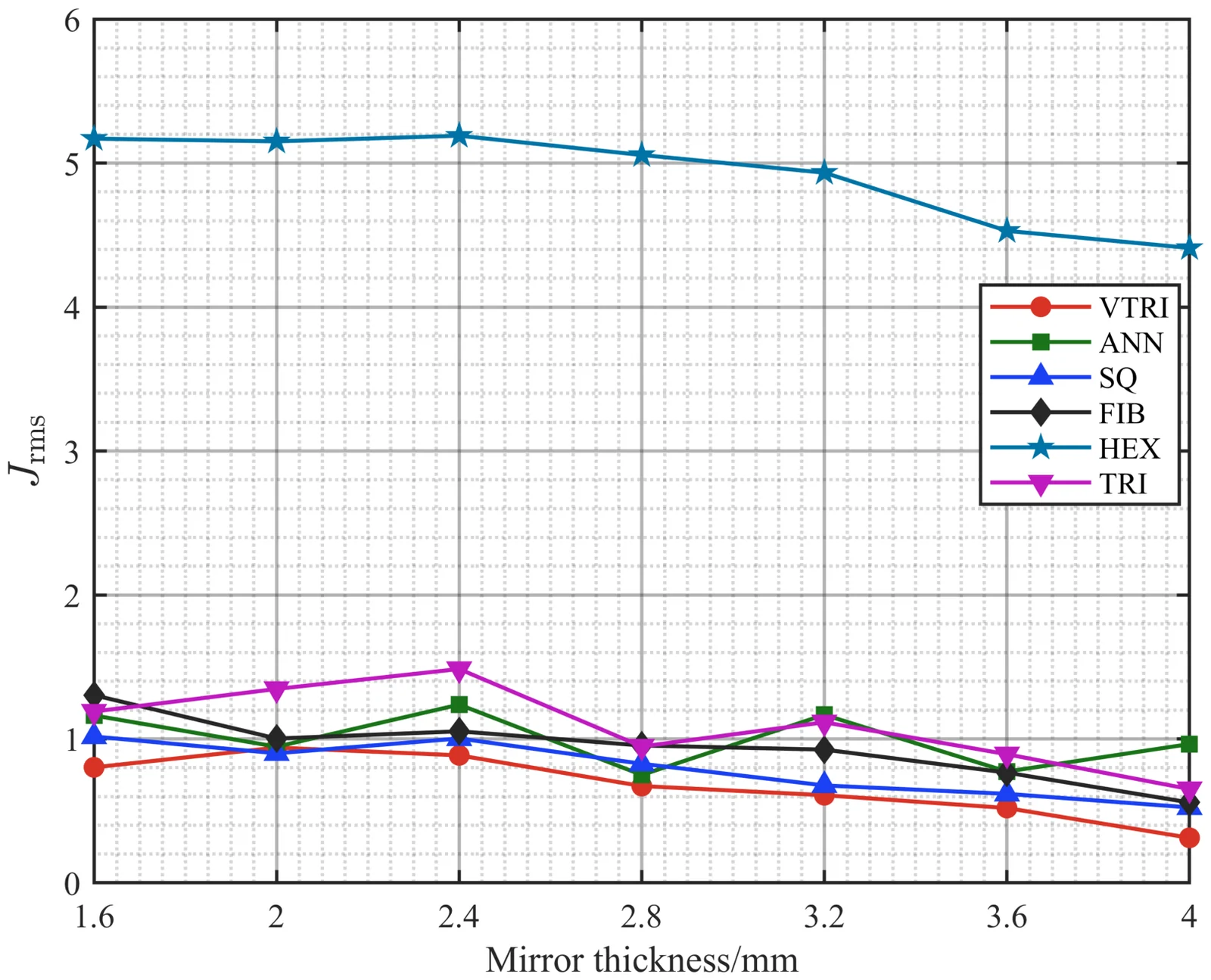
The weighted RMS residual of the six layouts as functions of mirror thickness.

The six layouts are also compared over the mirror-thickness range 1.6–4.0 mm using actuator-force limits sufficient to meet the correction requirement. As shown in Figure 21, VTRI maintains the lowest weighted RMS residual throughout this range, followed by SQ, FIB, ANN, TRI, and HEX. The residual of each layout changes only slightly with thickness. For a fixed support-pad diameter and boundary model, thickness therefore has little effect on the relative ranking of the layouts. Together with the influence-function results in Section 4.1, this trend indicates that thickness primarily scales the unit-force response amplitude and only weakly affects its spatial form. The differences in residual are consequently governed mainly by the ability of each layout to span the target modal space. VTRI provides the most consistent residual control across the full thickness range.

## 7. Discussion

The results demonstrate that active support optimization for an annular thin mirror cannot be reduced to increasing the number of actuators. Although actuator count sets an upper bound on spatial sampling, the radial coordinates, azimuthal phases, and local connectivity determine how effectively the influence-function matrix spans the target space. Support topology must therefore be treated as an independent design variable when both actuator count and force are constrained.

The proposed workflow is not restricted to the specific mirror considered here. For other mirror geometries, operating conditions, or boundary conditions, the equivalent-modeling strategy, surrogate construction, and optimization formulation can be repeated using the corresponding FE data and engineering constraints.

VTRI derives its advantage from combining a triangular skeleton with local nodal freedom. The skeleton provides regular nearest-neighbor connectivity and limits unsupported spans, whereas node motion adapts the radial and azimuthal sampling without changing the underlying topology. This combination reduces the directional bias associated with a fixed lattice. VTRI also retains comparatively large middle and tail singular values among the rank-preserving layouts, while achieving the lowest corrected RMS residual of all six layouts.

Six-sector rotational symmetry distinguishes VTRI from a fully free layout. It reduces the design dimension, provides a clear alignment datum, and facilitates actuator grouping. For the axial-gravity, radial-gravity, and low-order Annular Zernike targets *Z*_4_–*Z*_11_ considered here, this symmetry balances the sampling requirements of axisymmetric, two-fold, and three-fold modes. It may, however, restrict correction of strongly asymmetric high-order modes and localized high-spatial-frequency errors. The present conclusions are therefore most applicable to annular thin mirrors whose error budgets are dominated by gravity deformation and low-order aberrations.

VTRI effectively corrects the low-order modes *Z*_4_–*Z*_11_ and retains useful control authority over several mid- and high-order modes. Some higher-order modes, however, project only weakly onto the actuator influence-function space and consequently retain comparatively large residuals. More uniform high-order controllability would require either additional actuators or explicit inclusion of representative high-order modes in the layout-optimization objective.

The comparison isolates the effects of support geometry, influence-function coverage, and force constraints using the same mirror model, support-pad diameter, allowable region, minimum spacing, and actuator-force limit for all layouts. A detailed engineering design must additionally account for actuator architecture, flexural connectors, adhesive load transfer, local print-through, installation volume, and stress. In particular, pad diameter and connection stiffness affect the local influence-function shape, whereas the equivalent stiffness of the central flexure influences the low-order response. The optimized layout should therefore be verified using a complete optomechanical model.

Rotational reduction and Kriging interpolation substantially improve optimization efficiency by enabling rapid response-matrix construction for many candidate support locations. Their accuracy nevertheless depends on the quality of the FE samples, the fidelity of the equivalent boundary conditions, and the prediction accuracy of the Annular Zernike coefficients. Future interferometric measurements of single-actuator influence functions should be used to validate the surrogate at each Zernike order and to compare the singular-value spectra of the measured and predicted response matrices. Such tests would establish whether the surrogate preserves the modal-coverage characteristics of the physical support system.

The present conclusions are based on FE simulations and engineering experience with gravity-unloading systems for large mirrors and have not yet been validated experimentally for the mirror considered here. Future work will therefore develop a full-scale proof-of-principle prototype. The unit-force influence function of each actuator will be measured interferometrically and compared with the FE/surrogate prediction in Figure 9a and its Annular Zernike decomposition in Figure 9b. These comparisons will assess the amplitude and phase accuracy of the low-order modes *Z*_4_–*Z*_11_ and provide experimental validation of the response matrix *H*(*x*). Gravity-direction switching tests will be used to evaluate axial- and radial-gravity correction, and prescribed *Z*_4_–*Z*_11_ surface errors will be introduced under interferometric monitoring to compare measured and predicted corrected RMS residuals. In addition, the 0.5 N incremental actuator-force limit and constrained solution in Equation (14) assume ideal actuator behavior. Force–stroke calibration and force-resolution measurements will therefore be used to characterize the actual force–displacement relation and hysteresis and to quantify their effects on residual performance and force margin.

## 8. Conclusions

This study developed and evaluated an active support layout for a 660 mm aperture annular thin mirror with a 100 mm central aperture. The principal conclusions are as follows.

First, a unified optimization framework was formulated with the support coordinates as the primary design variables. The formulation combines residuals for gravity and low-order Annular Zernike targets with the allowable support region, minimum spacing, mesh-quality constraint, and actuator-force limit. Modal coverage is evaluated using the response matrix, singular-value spectrum, effective rank, and corrected RMS residual.

Second, a six-sector variable triangular layout was proposed. It uses a regular triangular grid as the initial topology, allows local node motion within the basic sector, and generates the full layout by 60° rotational replication. For the central-flexure-constrained mirror considered here, the optimized VTRI layout demonstrates favorable modal controllability and constrained correction capability, with an effective rank of 35 and the lowest mean projection and regularization residuals among the six layouts considered.

Third, the approximate rotational symmetry of the mirror reduced the unit-force response model from W(R,θ,h) to W(R,h). A Kriging surrogate trained on the FE samples reproduced the Annular Zernike coefficients, response-matrix singular values, and corrected RMS residuals obtained by direct FE analysis with high accuracy.

Fourth, at the same support count and under identical geometric and force constraints, the optimized 36-point VTRI layout effectively corrected axial gravity, radial gravity, and the low-order Annular Zernike modes *Z*_4_–*Z*_11_. Every corrected RMS residual satisfied the 12.66 nm requirement. Relative to ANN, TRI, SQ, HEX, and FIB, VTRI achieved the lowest composite objective-function value and mean RMS residual.

Finally, the present assessment focuses on layout geometry, modal coverage, and force constraints. Future work should incorporate detailed actuator and flexure models, adhesive-layer load transfer, local stress and print-through, assembly errors, and measured influence functions. Force reconstruction and fault-tolerant control under actuator failure should also be investigated to establish the engineering reliability of the proposed system.

## Figures and Tables

**Table 1:** Target surfaces and evaluation requirements for support-layout optimization.

Category	Target Surface	Requirement
Surface maintenance	Axial gravity, *G_z_*	RMS ≤ 12.66 nm
Surface maintenance	Radial gravity, *G_y_*	RMS ≤ 12.66 nm
Correction target	189 nm *Z*_4_, defocus	RMS ≤ 12.66 nm
Correction target	316 nm *Z*_5_, 45° astigmatism	RMS ≤ 12.66 nm
Correction target	316 nm *Z*_6_, 0° astigmatism	RMS ≤ 12.66 nm
Correction target	189 nm *Z*_7_, 90° coma	RMS ≤ 12.66 nm
Correction target	189 nm *Z*_8_, 0° coma	RMS ≤ 12.66 nm
Correction target	127 nm *Z*_9_, trefoil	RMS ≤ 12.66 nm
Correction target	127 nm *Z*_10_, trefoil	RMS ≤ 12.66 nm
Correction target	189 nm *Z*_11_, primary spherical aberration	RMS ≤ 12.66 nm

Note: All RMS values in this paper denote the mirror surface-figure error evaluated after removing the rigid-body displacement terms (piston and tilt, Z1–Z3). The RMS criterion of 12.66 nm and the Z4–Z11 amplitudes of 127–316 nm are representative correction requirements for high-resolution optical remote-sensing systems. These values describe the compensation demands of mirror deformation and low-order system aberrations allocated to the active primary mirror [4,8,36,37]. For different optical systems, the allowable residual error and correction amplitudes should be determined according to the specific system requirements.

**Table 2:** Material parameters of candidate mirror materials.

Material	*E* (GPa)	Poisson Ratio	Density (g/cm^3^)	Allowable Stress (MPa)
ULE glass	68.0	0.24	2.20	5
Zerodur glass ceramic	88.7	0.24	2.53	8
Silicon carbide	392	0.25	3.05	40

**Table 3:** Parameterization characteristics of different support layouts.

Layout	Symmetry	Variables (36 Supports)	Parameterization
TRI	Six-fold	2	Lattice scale and rotation
SQ	Four-fold	2	Lattice scale and rotation
HEX	Six-fold	2	Lattice scale and rotation
ANN	Circular	6	Ring radii and phase offsets
FIB	No strict symmetry	4	Initial phase and radial parameters
VTRI	Six-fold	12	Movable sector nodes with triangular adjacency

**Table 4:** Target and corrected RMS values for the gravity, low-order aberration, and combined-aberration cases.

Target	Target RMS (nm)	Corrected RMS (nm)	Requirement (nm)
Axial gravity	68.17	1.46	≤12.66
Radial gravity	9.11	1.89	≤12.66
Defocus (*Z*_4_)	189	1.60	≤12.66
45° astigmatism (*Z*_5_)	316	2.88	≤12.66
0° astigmatism (*Z*_6_)	316	2.28	≤12.66
90° coma (*Z*_7_)	189	2.11	≤12.66
0° coma (*Z*_8_)	189	2.84	≤12.66
Trefoil (*Z*_9_)	127	2.04	≤12.66
Trefoil (*Z*_10_)	127	2.63	≤12.66
Spherical aberration (*Z*_11_)	189	1.71	≤12.66
Overlay term	245	3.06	≤12.66

**Table 5:** Correction performance of layouts with different symmetry orders, numbers of design variables, and minimum-spacing constraints.

Layout	Objective Value	Mean RMS (nm)	Number of Variables
VTRI	0.34	2.15	12
TRI	0.54	2.36	2
6-array-80	0.38	2.20	12
6-array-50	0.28	1.60	12
4-array-80	0.49	2.99	18
4-array-50	0.38	2.24	18
3-array-80	0.60	3.18	24
3-array-50	0.39	2.20	24
2-array-80	0.56	2.72	36
2-array-50	0.59	2.88	36
Free-80	0.72	3.02	72
Free-50	0.84	3.35	72

**Table 6:** Correction-performance metrics for six optimized 36-point layouts.

Layout	Composite Objective	Mean RMS (nm)	Mean Projection Residual	Mean Regularization Residual	Effective Singular-Value Condition Number	Rank
VTRI	0.34	2.15	0.010158	0.563	5710.8	35
SQ	0.38	2.27	0.010161	0.597	4889.6	35
ANN	0.77	3.12	0.339	0.618	1600.4	27
HEX	4.28	4.83	0.214	0.663	924.44	31
TRI	0.54	2.36	0.055	0.589	3993.9	34
FIB	0.45	2.35	0.010161	0.624	4862.6	35

Note: The mean projection residual is defined as ${\bar{R}}_{\mathrm{proj}}=\frac{1}{33}\sum_{j=4}^{36}\frac{{∥z_{j}-Hf_{\mathrm{proj},j}∥}_{2}}{{∥z_{j}∥}_{2}}$, where $z_{j}$ is the unit-normalized vector of the *j*th Annular Zernike target mode and $f_{\mathrm{proj},j}$ is the corresponding actuator-force vector obtained by projection onto the truncated effective response subspace. The calculation covers the 33 modes from *Z*_4_ to *Z*_36_, and ${\bar{R}}_{\mathrm{proj}}$ is their equally weighted arithmetic mean of the relative projection residuals. The mean regularization residual is defined as ${\bar{R}}_{\mathrm{reg}}=\frac{1}{33}\sum_{j=4}^{36}\frac{{∥z_{j}-Hf_{\mathrm{reg},j}∥}_{2}}{{∥z_{j}∥}_{2}}$, where $f_{\mathrm{reg},j}$ is the actuator-force vector obtained using the truncated, regularized damped least-squares solution; ${\bar{R}}_{\mathrm{reg}}$ is the equally weighted arithmetic mean of the relative regularization residuals over the same 33 modes. Effective rank *r* is evaluated using the threshold $\sigma_{i}/\sigma_{1}>10^{-4}$, and the reported condition number is $\kappa =\sigma_{1}/\sigma_{r}$, where $\sigma_{1}$ is the largest singular value and $\sigma_{r}$ is the singular value at the effective-rank cutoff.

## Data Availability

The data presented in this study are available in the article and from the corresponding author upon reasonable request.

## Abbreviations

ANN	Annular layout
CMA-ES	Covariance matrix adaptation evolution strategy
FE	Finite element
FIB	Fibonacci-spiral layout
HEX	Hexagonal layout
RMS	Root mean square
SQ	Square layout
TRI	Triangular layout
VTRI	Variable triangular layout
